# Associations of Ambient Air Pollution and Road-Related Environmental Characteristics with Cardiometabolic Health: The Role of Walking

**DOI:** 10.3390/toxics14100920

**Published:** 2026-10-17

**Authors:** Ester Cerin, Miguel Alvarado-Molina, Muhammad Akram, Yih-Kai Chan, Jonathan E. Shaw, Luke D. Knibbs, Anthony Barnett

**Affiliations:** 1 Mary Mackillop Institute for Health Research, Australian Catholic University, Melbourne, VIC 3000, Australia; maalvaradom@proton.me (M.A.-M.); yihkai.chan@acu.edu.au (Y.-K.C.); anthony.barnett@acu.edu.au (A.B.); 2 School of Public Health, The University of Hong Kong, 7 Sassoon Rd., Sandy Bay, Hong Kong; 3 Baker Heart and Diabetes Institute, Melbourne, VIC 3004, Australia; jonathan.shaw@baker.edu.au; 4 Hudson Institute of Medical Research, Monash University, Clayton, VIC 3168, Australia; 5 School of Public Health and Preventive Medicine, Monash University, Melbourne, VIC 3004, Australia; 6 School of Life Sciences, La Trobe University, Melbourne, VIC 3086, Australia; 7 Sydney School of Public Health, The University of Sydney, Camperdown, NSW 2050, Australia; l.knibbs@uq.edu.au; 8 Public Health Research Analytics and Methods for Evidence, Public Health Unit, Sydney Local Health District, Camperdown, NSW 2050, Australia; 9 School of Public Health, The University of Queensland, Herston, QLD 4006, Australia

**Keywords:** ambient air pollution, PM_2.5_, nitrogen dioxide, road density, walking, active transport, cardiometabolic health, built environment

## Abstract

Ambient air pollution and road environments may have contrasting associations with walking and cardiometabolic health. We examined these relationships in 4141 middle-aged and older adults from the third wave of the Australian Diabetes, Obesity and Lifestyle Study. Generalized additive mixed models estimated associations of residential PM_2.5_, NO_2_, total and major road density, and distance to the nearest busy road with cardiometabolic indicators and walking behaviors. Benjamini–Hochberg false discovery rate adjustment was applied within defined test families. Cross-sectional patterns involving walking were evaluated using an FDR-adjusted joint-significance criterion. PM_2.5_ was positively associated with diastolic blood pressure and non-linearly associated with systolic blood pressure; NO_2_ was non-linearly associated with systolic blood pressure. Total road density was associated with lower HDL cholesterol and higher triglycerides; major road density was associated with higher triglycerides and non-linearly with HbA1c. Distance to the nearest busy road was non-linearly associated with HbA1c. Higher road densities were associated with less total walking time among walkers, whereas higher PM_2.5_ and NO_2_ were associated with greater participation in walking for transport. Joint-significance patterns involved waist circumference and lipids. These findings highlight the importance of considering environmental exposures and walking together but do not establish temporal ordering or causal mediation.

## 1. Introduction

Urban transport environments are important determinants of population health because they shape mobility, environmental exposures and access to destinations of daily living [1,2,3,4]. These environments can affect cardiometabolic (CMB) health through potentially opposing pathways. Compact and connected neighborhoods may facilitate walking and active transport, thereby increasing health-enhancing physical activity, while traffic and other urban emission sources contribute to ambient air pollution, exposure to which may adversely affect health [5]. Understanding these interconnected environmental and behavioral pathways is important for designing urban environments that support mobility while minimizing harmful environmental exposures.

Long-term exposure to ambient air pollution has been associated with a range of adverse CMB outcomes [6,7,8,9,10,11,12,13,14,15]. Fine particulate matter with an aerodynamic diameter of ≤2.5 μm (PM_2.5_) and nitrogen dioxide (NO_2_) are commonly investigated in this context, although they differ in their sources and specificity as indicators of traffic-related air pollution [16]. Road traffic is an important source of both pollutants in urban areas. However, ambient PM_2.5_ originates from multiple primary and secondary sources and, therefore, cannot be considered a traffic-specific indicator. In contrast, NO_2_ generally shows greater spatial specificity to road-traffic emissions in urban environments, although it is not exclusively traffic-derived [16]. Long-term exposure to PM_2.5_ and NO_2_ has been associated with increased adiposity [12,13], elevated blood pressure [8,14], dyslipidemia [10,11,15] and impaired glucose metabolism [12]. Traffic-related exposures have also been characterized using road-based indicators, including road density and residential proximity to major or busy roads [17,18]. These indicators provide complementary information about the traffic environment but do not directly quantify individual exposure to specific air pollutants.

An important complication is that air-pollution and road-based indicators can also be associated with characteristics of the urban environments that support physical activity. Connected street networks, higher road density and proximity to major activity corridors are often found in areas providing greater access to destinations, and destination accessibility is an important determinant of walking, particularly walking for transport [19,20,21]. Walking, in turn, is associated with better CMB health [22,23,24]. Consequently, associations between traffic- and road-related environmental characteristics and health may reflect a combination of potentially harmful pollution-related pathways and beneficial behavioral pathways. Even spatial variation in modeled air-pollutant concentrations may partly correlate with urban form and destination accessibility. Failure to account for these interrelationships may therefore obscure the pathways through which urban environmental exposures influence health.

These issues may be particularly relevant in relatively low-pollution settings such as Australian cities, where ambient concentrations of air pollutants are generally lower than those reported in many North American, European and Asian cities [25]. Previous Australian studies illustrate the complexity of these relationships. Modeled annual average concentrations of NO_2_ and PM_2.5_ were positively associated with walking for transport and, unexpectedly, with better cognitive health among middle-aged and older adults [26]. Similarly, modeled NO_2_ concentrations were positively associated with walking for transport but were not adversely associated with health-related quality of life [19]. Such findings do not necessarily indicate beneficial effects of air pollution. Rather, they suggest that spatially modeled pollutant concentrations and road-related indicators may covary with characteristics of the urban environment that facilitate access to destinations and active travel. Disentangling these environmental and behavioral pathways is therefore necessary for interpreting associations between air pollution, traffic-related environmental characteristics and health.

Previous analyses of the third wave of the Australian Diabetes, Obesity and Lifestyle (AusDiab3) Study have examined selected associations among neighborhood environmental attributes, modeled ambient air pollution, physical activity and CMB or other health outcomes [12,19,22,26]. The present study builds on this work by addressing a distinct question: whether ambient air-pollution indicators and road-related environmental characteristics show different patterns of association with CMB health and whether their associations with total walking and walking for transport are consistent with hypothesized behavioral pathways. We additionally examine the robustness of findings involving road density across alternative neighborhood spatial specifications. More broadly, relatively few studies have simultaneously considered ambient air pollution, road-related environmental characteristics and behavioral pathways involving walking [22]. Examining these environmental indicators and behaviors together may provide a more complete understanding of how interrelated characteristics of urban environments are associated with CMB health.

Accordingly, the present study examined cross-sectional associations of two ambient air-pollution indicators—annual average residential concentrations of PM_2.5_ and NO_2_—and three road-related environmental indicators—total road density, major road density and distance to the nearest busy road—with multiple CMB health indicators among middle-aged and older adults living in Australian cities. We additionally examined whether patterns of associations involving total walking and walking for transport were consistent with hypothesized behavioral pathways. We hypothesized that higher concentrations of ambient air pollutants and greater road/traffic intensity or proximity, as reflected by the road-related indicators, would be associated with less favorable CMB health. However, because some road-related characteristics and spatial patterns of air pollution may covary with destination accessibility, we also hypothesized that some environmental indicators would be positively associated with walking and that greater walking would be associated with more favorable CMB health. Given the cross-sectional design, these analyses were intended to identify combinations of associations consistent with the proposed behavioral relationships rather than to establish temporal ordering, indirect effects or causal mediation.

## 2. Materials and Methods

### 2.1. Study Design and Sample

This study was a cross-sectional analysis of data from the third wave of the Australian Diabetes, Obesity and Lifestyle (AusDiab) Study, a longitudinal population-based cohort study of Australian adults [27]. In total, 11,247 persons participated in the baseline assessments, 6527 continued during the second wave, and 4614 returned for the third wave. Participants were recruited from 42 randomly selected census collection districts across Australia (Figure S1.1). The present analyses used data from the third wave of AusDiab (AusDiab3) collected in 2011–2012, for which the environmental exposure measures required for this study were available. The analytical sample comprised 4141 participants residing within Significant Urban Areas, defined by the Australian Bureau of Statistics as urban areas with populations >10,000 [28]. Further details on participants’ recruitment and assessment protocols are provided elsewhere [27,29]. The study was conducted according to the guidelines of the Declaration of Helsinki and approved by the Alfred Hospital Ethics Committee, Melbourne, Australia [ref. no 39/11; 2 March 2011]. All participants provided written informed consent.

### 2.2. Environmental Exposure Measures

Annual average concentrations of PM_2.5_ (μg/m^3^) and NO_2_ (ppb) were estimated at each participant’s residential address using national satellite-based land-use regression models [30,31,32]. The models combined spatial predictors of annual average pollutant concentrations at fixed-site monitors with time-varying satellite-derived information to estimate concentrations at unmeasured locations and were used to predict exposure at the time of AusDiab3. The NO_2_ model explained 81% of spatial variability in annual NO_2_ concentrations (RMSE: 1.4 ppb), while the PM_2.5_ model explained 63% of spatial variability in annual PM_2.5_ concentrations (RMSE: approximately 1 μg/m^3^) [30,31,32]. PM_2.5_ was considered an indicator of ambient air pollution rather than a specific proxy for transport-related air pollution (TRAP) because ambient PM_2.5_ originates from multiple primary and secondary sources. NO_2_ was also considered an ambient air-pollution indicator but has greater spatial specificity to road-traffic emissions in urban environments.

Road-related environmental indicators were derived from the 2012 Australian road network provided by Geoscape Australia (formerly PSMA Australia) using ArcGIS V.10.5 (ESRI, Redlands, CA, USA) [33]. Total road density and major road density (m/km^2^) were calculated within Euclidean buffers of 200 m, 500 m and 1 km around each participant’s residential address. Road-density measures within the 1 km buffer were used in the principal analyses presented in the main manuscript because this spatial scale approximates a plausible neighborhood walking range among middle-aged and older adults [34,35]. Corresponding analyses using 200 m and 500 m buffers were conducted to examine the robustness of findings across spatial scales and are reported in the Supplementary Material. Aerial distance (m) from each participant’s residential address to the nearest busy road was also calculated as a residential indicator of proximity to road traffic.

### 2.3. Cardiometabolic (CMB) Health (Outcomes)

CMB health outcomes considered in our study are waist circumference (cm) as an indicator of adiposity levels; systolic and diastolic blood pressure (mmHg) as indicators of blood pressure measured in accordance with the World Health Organization (WHO) guidelines; high- and low-density lipoprotein (HDL/LDL, mmol/L) together with triglycerides (mmol/L) as indicators of dyslipidemia, and glycated hemoglobin or HbA1c (mmol/mol), which indicates the glucose levels in the bloodstream over the previous three to four months [36]. Standardized measurement protocols were used, which are detailed elsewhere [27,29].

### 2.4. Walking Behaviors

Two self-reported walking measures from AusDiab3, adapted from the Active Australia Survey [37], were examined as hypothesized behavioral pathways linking environmental indicators with CMB health: total walking and walking for transport. Both measures referred to walking during the previous week. Total walking was assessed as the total time spent walking for any purpose (min/week), whereas walking for transport was assessed as the frequency of walking to get to and from places (times/week).

Because both measures contained a substantial proportion of zero values corresponding to reported non-participation in the respective walking behavior, we used a two-part (hurdle-type) modeling approach. This approach distinguishes between two potentially different behavioral processes: (1) whether an individual engaged in the walking behavior and (2) the amount or frequency of walking among those who engaged in the behavior. Accordingly, total walking was represented by engagement in any walking during the previous week (yes/no) and total walking time (min/week) among those reporting any walking. Walking for transport was represented by engagement in any walking for transport (yes/no) and frequency of walking for transport (times/week) among those reporting this behavior. Total walking and walking for transport were examined in separate analyses because walking for transport contributes to total walking and the two measures should not be considered independent behaviors.

### 2.5. Covariates

Minimal sufficient sets of potential confounders were identified using a directed acyclic graph (DAG) informed by previous evidence and substantive knowledge (Figure S2.1). Models were adjusted for individual-level sociodemographic characteristics, health-related behaviors (e.g., smoking) and residential self-selection, as well as neighborhood-level characteristics that could confound associations between the environmental indicators and CMB health outcomes. These included area-level socioeconomic status, population density, land use and transport-accessibility indicators. The covariates and rationale for their inclusion are described in Supplementary Material S2, with the hypothesized relationships among environmental indicators, covariates, walking behaviors and CMB health outcomes represented in Figure S2.1.

### 2.6. Statistical Analysis

Generalized additive mixed models (GAMMs) were used to estimate associations of the environmental indicators with CMB health outcomes and walking behaviors. GAMMs were fitted using the ‘mgcv’ package (version 1.9-4) [38] in R version 4.6.1 [39] and included random intercepts at the Statistical Area Level 1 (SA1) to account for spatial clustering of participants. GAMMs were selected because they accommodate outcomes with different distributional assumptions and allow potentially non-linear associations to be modeled using smooth terms. Covariate adjustment sets were informed by the DAG described in Supplementary Materials S2 and S3. The functional form of associations with environmental indicators was evaluated by comparing models containing linear and smooth terms. Evidence supporting a non-linear association was considered present when the model containing the smooth term had an Akaike Information Criterion (AIC) at least 10 units lower than the corresponding model containing the linear term; further details are provided in Supplementary Material S3.

We first estimated associations of each ambient air-pollution and road-related environmental indicator with each CMB health outcome. Primary analyses of road-density indicators used measures defined within 1 km residential buffers, consistent with the hypothesized neighborhood walking range described in Section 2.2. Analyses using corresponding 200 m and 500 m road-density measures were conducted as spatial scale sensitivity analyses and are reported in the Supplementary Material. Interactions between environmental indicators and the medication corresponding to each CMB outcome were included where applicable (lipid-lowering medication for lipid outcomes, antihypertensive medication for blood-pressure outcomes and diabetes medication for HbA1c).

We subsequently examined whether patterns of associations involving walking were consistent with the hypothesized behavioral pathways described in the Introduction. For each environmental indicator, we examined (1) its association with each walking measure and (2) the association of the walking measure with each CMB health outcome after adjustment for the environmental indicator. Following the joint-significance approach [40], we identified instances in which both component associations were statistically supported. Following False Discovery Rate (FDR) correction, the joint-significance criterion was considered met only when both component associations had FDR-adjusted *q*-values < 0.05. These were interpreted as cross-sectional patterns consistent with the hypothesized behavioral relationships involving walking, rather than as evidence of an indirect effect or mediation.

The distributions of the walking measures were characterized by a point mass at zero and positively distributed values among participants reporting the respective behavior. We therefore used a two-part modeling strategy, analogous to the decomposition underlying hurdle models, to distinguish associations with participation from associations with the amount or frequency of walking conditional on participation. The two components were estimated separately to retain the flexibility of GAMMs for modeling potentially non-linear associations and spatial clustering. Engagement in total walking and walking for transport (yes/no) was modeled using GAMMs with binomial variance and logit link functions. Among participants reporting the respective behavior, positive total walking time (min/week) was modeled using a Gamma distribution with a logarithmic link, while positive frequency of walking for transport (times/week) was modeled using a negative binomial distribution with a logarithmic link. Total walking and walking for transport were examined in separate sets of models.

For CMB health outcomes, GAMMs with Gaussian variance and identity link functions were used for waist circumference, systolic and diastolic blood pressure, HDL cholesterol and LDL cholesterol. Triglycerides and HbA1c were modeled using Gamma variance and logarithmic link functions. Estimates from models with logit links are reported as odds ratios (ORs) and estimates from models with logarithmic links are exponentiated and reported as ratios of expected outcome values, with corresponding 95% confidence intervals (95% CI).

Missing data were handled using multiple imputation by chained equations. Twenty imputed datasets were created using the mice package version 3.19.0 [41] in R version 4.6.1 [39]. Regression coefficients and their standard errors were combined across imputed datasets using Rubin’s rules. For associations represented by smooth terms, overall statistical significance across the multiply imputed datasets was evaluated using a D1 multivariate Wald test based on Rubin’s combining rules, jointly testing the coefficients representing the smooth term. Fitted exposure–response curves from all 20 imputed datasets were plotted to display the consistency of the estimated functional form across imputations.

To account for multiple testing, the Benjamini–Hochberg procedure was used to control the FDR at 5% within conceptually related families of tests. For associations between environmental indicators and CMB health, a separate FDR family was defined for each CMB outcome and comprised tests of the five environmental indicators. The same outcome-specific five-indicator FDR correction was applied separately to environmental indicator–CMB associations estimated in models additionally adjusted for total walking or walking for transport. For associations between environmental indicators and walking, separate FDR families were defined for each walking measure and each component of the corresponding two-part model. Thus, participation in total walking, non-zero total walking time, participation in walking for transport, and non-zero walking-for-transport frequency were each treated as separate families, with each family comprising tests of the five environmental indicators. For associations between walking and CMB health, separate FDR families were defined for each combination of walking measure, model component and CMB outcome, with each family comprising estimates from models adjusted separately for each of the five environmental indicators. The same FDR-family structure was applied independently to the primary 1 km spatial specification and to each of the 500 m and 200 m sensitivity specifications, allowing like-for-like comparison of FDR-adjusted findings across spatial scales. Effect estimates, 95% confidence intervals and the consistency of associations across related analyses and spatial specifications were also considered in interpretation.

Statistical tests were two-sided. Unadjusted *p*-values and Benjamini–Hochberg FDR-adjusted *q*-values were reported, with *q* < 0.05 used to identify statistically supported associations after accounting for multiple testing.

## 3. Results

### 3.1. Descriptive Statistics

Participants had a mean age of 61.1 years (SD = 11.4), and 55.2% were female (Table 1). Just over half were in paid employment and 7.0% were current smokers. Over 80% reported any walking during the previous week and, among participants reporting walking (*n* = 3336), mean total walking was 209.7 min/week (SD = 197.6). Nearly 30% reported any walking for transport and, among those reporting this behavior (*n* = 1206), mean frequency was 4.6 times/week (SD = 5.08). Average total walking was 169 min/week, while frequency of walking for transport was 1.4 times/week.

Descriptive statistics for the environmental indicators and neighborhood-level covariates related to the primary analyses are presented in Table 2, while those used for sensitivity analyses are reported in the Supplementary Material (Table S4.1). Mean annual residential concentrations of PM_2.5_ and NO_2_ were 6.3 μg/m^3^ (SD = 1.7) and 5.5 ppb (SD = 2.1), respectively. Mean total and major road densities within the 1 km residential buffer were 9576 m/km^2^ (SD = 3404) and 1314 m/km^2^ (SD = 895), respectively, while mean distance to the nearest busy road was 457 m (SD = 498), ranging from 2 m to 3500 m.

Correlations among the environmental indicators ranged from weak to strong (Tables S5.1–S5.3). The strongest correlations were observed between major road density indicators and distance to the nearest busy road, while total and major road density indicators were moderately positively correlated. PM_2.5_ and NO_2_ generally showed weak-to-moderate correlations with the road-related environmental indicators.

### 3.2. Associations of Ambient Air Pollution and Road-Related Environmental Indicators with Cardiometabolic Health Outcomes

**Figure 1:**
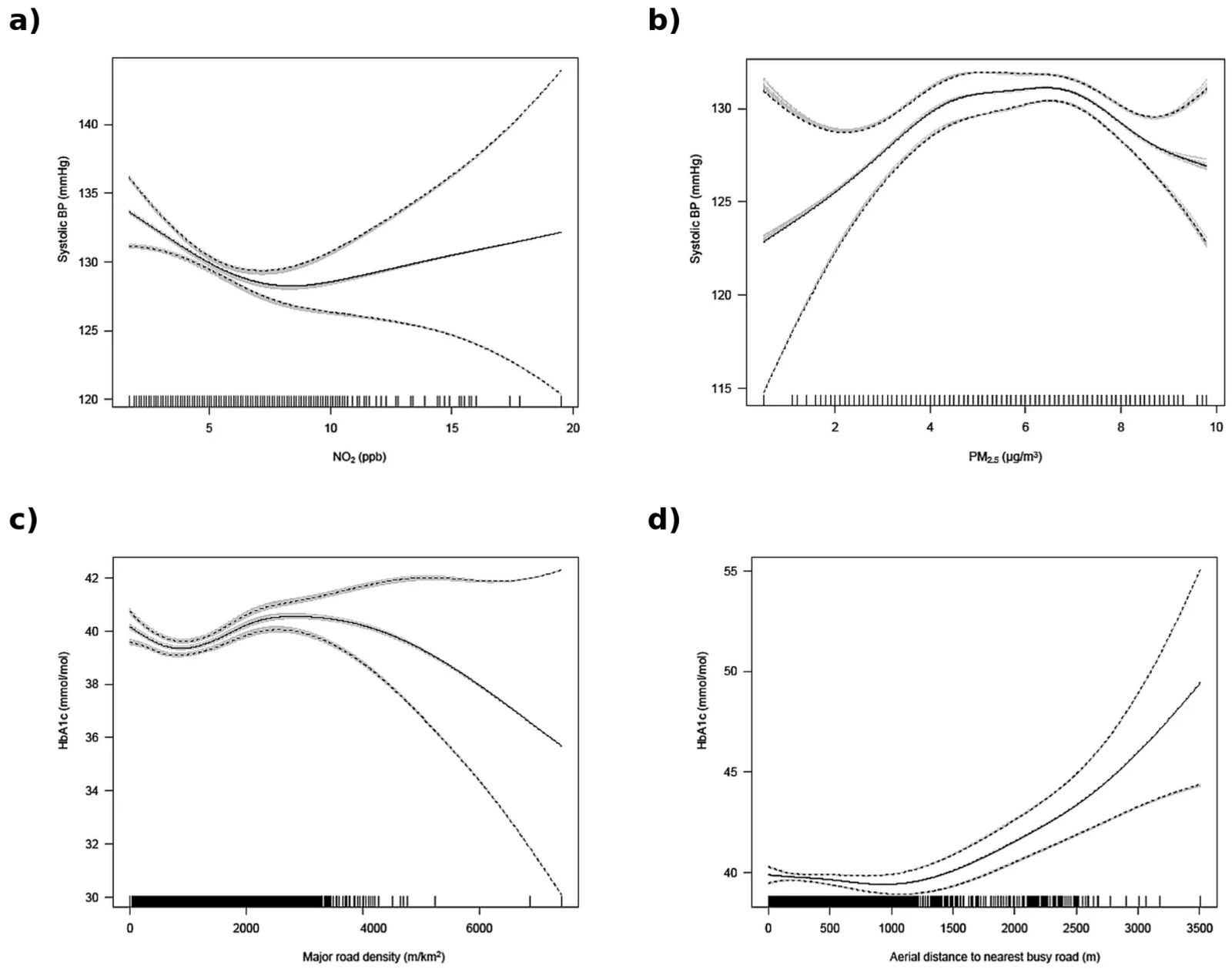
Non-linear associations of: (**a**) NO_2_ with systolic blood pressure, (**b**) PM_2.5_ with systolic blood pressure, (**c**) major road density with HbA1c, and (**d**) aerial distance to the nearest busy road with HbA1c. Curves represent fitted associations from each of the 20 imputed datasets; dotted lines are 95% CIs.

Associations of the environmental indicators with CMB health outcomes from the primary analyses are presented in Table 3. Among the ambient air-pollution indicators, PM_2.5_ showed a positive association with diastolic blood pressure (*b* = 0.373; 95% CI: 0.138, 0.609; *p* = 0.002; *q* = 0.010) and a non-linear relationship with systolic blood pressure (*p* < 0.001; *q* < 0.001; Figure 1b). NO_2_ showed a non-linear association with systolic blood pressure (*p* = 0.002; *q* = 0.005; Figure 1a).

Among the road-related environmental indicators, higher total road density within 1 km of participants’ residences was associated with lower HDL cholesterol (*b* = −0.019; 95% CI: −0.031, −0.007; *p* = 0.001; *q* = 0.005) and higher triglyceride levels (e*ᵇ* = 1.032; 95% CI: 1.015, 1.049; *p* < 0.001; *q* < 0.001). Higher major road density within 1 km was also associated with higher triglyceride levels (e*ᵇ* = 1.042; 95% CI: 1.017, 1.067; *p* < 0.001; *q* < 0.001) and showed a non-linear association with HbA1c (*p* = 0.001; *q* = 0.003; Figure 1c). Distance to the nearest busy road was non-linearly associated with HbA1c (*p* < 0.001; *q* = 0.001; Figure 1d), with HbA1c tending to decrease at shorter distances and increase at longer distances.

Results from the spatial scale sensitivity analyses were generally consistent with those of the primary analyses, although several associations varied across spatial specifications (Tables S6.1 and S6.2). The non-linear associations of NO_2_ and PM_2.5_ with systolic blood pressure and the positive association of PM_2.5_ with diastolic blood pressure remained statistically supported at both 200 m and 500 m. In addition, NO_2_ was inversely associated with diastolic blood pressure in the 200 m (*b* = −0.348; 95% CI: −0.579, −0.117; *p* = 0.003; *q* = 0.009) and 500 m (*b* = −0.325; 95% CI: −0.567, −0.083; *p* = 0.009; *q* = 0.023) specifications, but not in the primary 1 km analysis. For the road-related indicators, the associations of total road density with lower HDL cholesterol and higher triglycerides, and of major road density with higher triglycerides, remained statistically supported at 500 m but not at 200 m. The non-linear association of major road density with HbA1c was observed only in the primary 1 km analysis. In contrast, the non-linear association between distance to the nearest busy road and HbA1c was consistently observed across all three spatial specifications.

### 3.3. Cross-Sectional Association Patterns Involving Walking

**Figure 2:**
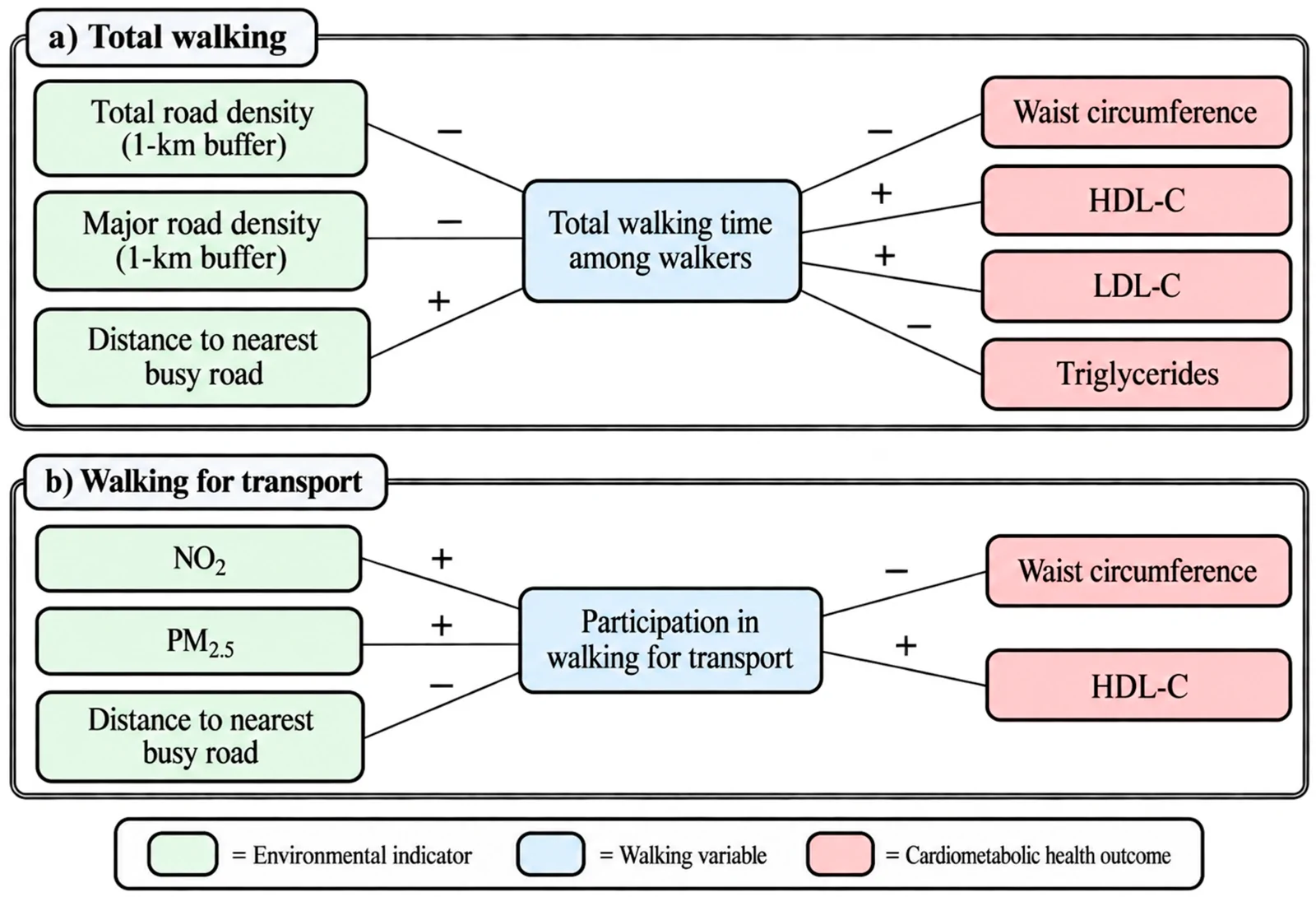
Schematic summary of cross-sectional association patterns among environmental indicators, walking and cardiometabolic health outcomes that met the FDR-adjusted joint-significance criterion in the primary analyses (1 km road-density measures and corresponding covariate specifications) of (**a**) total walking and (**b**) walking for transport. Lines without arrowheads indicate statistically supported component associations and do not represent temporal ordering or causal mediation. + and − indicate positive and negative associations, respectively. HDL-C—high-density lipoprotein cholesterol; LDL-C—low-density lipoprotein cholesterol; HbA1c—glycated hemoglobin; FDR—false discovery rate.

Patterns of associations involving total walking and walking for transport were examined to identify combinations of environmental indicator–walking and walking–CMB health associations that met the joint-significance criterion (Tables S7.1 and S7.2; Figure 2). These analyses identify co-occurring component associations and do not establish temporal ordering or causal mediation. In the primary 1 km analyses, higher total road density was associated with less total walking time among participants who reported any walking (e*^b^* = 0.939; 95% CI: 0.913, 0.966; *p* < 0.001; *q* < 0.001). Higher major road density was also associated with less total walking time (e*^b^* = 0.953; 95% CI: 0.912, 0.994; *p* = 0.027; *q* = 0.045), whereas greater distance to the nearest busy road was associated with more total walking time (e*^b^* = 1.010; 95% CI: 1.003, 1.017; *p* = 0.006; *q* = 0.015). NO_2_ and PM_2.5_ were not significantly associated with either measure of total walking.

Participation in total walking was consistently associated with smaller waist circumference (*b* = −1.797 to −1.774; all *p*s = 0.001; all *q*s = 0.001) and lower HbA1c (all e*^b^* = 0.986; *p*s = 0.024 to 0.030; all *q*s = 0.030) in models including different environmental indicators. However, because none of the environmental indicators were associated with participation in total walking after FDR adjustment, no association pattern involving total walking participation met the FDR-adjusted joint-significance criterion (see Table S7.1) and such patterns are therefore not included in Figure 2a but shown in Table S7.1.

Among participants reporting any walking, greater total walking time was consistently associated with smaller waist circumference (all *b*s = −0.006 per min/week; all *p*s < 0.001; all *q*s < 0.001), higher HDL-C (all *b*s = 0.0002; all *p*s < 0.001; all *q*s < 0.001), higher LDL-C (all *b*s = 0.0002; *p* = 0.002 to 0.003; all *q*s = 0.003) and lower triglycerides (e*^b^* = 0.9998 to 0.9999; *p* = 0.012 to 0.026; *q* = 0.021 to 0.026), with estimates varying little according to the environmental indicator included in the model. In combination with the environmental indicator–walking associations described above, the joint-significance criterion was met for association patterns involving total road density, major road density, or distance to the nearest busy road; total walking time; and these CMB outcomes (Figure 2a; Table S7.1).

A different pattern was observed for walking for transport. Higher NO_2_ (OR = 1.106; 95% CI: 1.046, 1.170; *p* < 0.001; *q* < 0.001) and PM_2.5_ (OR = 1.116; 95% CI: 1.057, 1.179; *p* < 0.001; *q* < 0.001) concentrations were associated with greater odds of participating in walking for transport, whereas greater distance to the nearest busy road was associated with lower odds of participation (OR = 0.979; 95% CI: 0.961, 0.998; *p* = 0.029; *q* = 0.048). Neither total nor major road density was significantly associated with participation in walking for transport. None of the environmental indicators was significantly associated with walking-for-transport frequency among participants reporting this behavior (Table S7.2).

Participation in walking for transport was associated with smaller waist circumference in the corresponding NO_2_ (*b* = −2.147 cm; 95% CI: −3.656, −0.638; *p* = 0.005; *q* = 0.006), PM_2.5_ (*b* = −2.195 cm; 95% CI: −3.703, −0.687; *p* = 0.004; *q* = 0.006) and distance-to-busy-road (*b* = −2.172 cm; 95% CI: −3.682, −0.663; *p* = 0.005; *q* = 0.006) models. It was also associated with higher HDL-C, with estimates ranging from *b* = 0.058 to 0.059 mmol/L (*p* = 0.011 to 0.013; all *q*s = 0.013). Consequently, the joint-significance criterion was met for association patterns involving NO_2_ or PM_2.5_, participation in walking for transport, and waist circumference or HDL-C. It was also met for corresponding patterns involving distance to the nearest busy road, for which greater distance was associated with lower participation in walking for transport (Figure 2b; Table S7.2). No association patterns involving walking-for-transport frequency met the joint-significance criterion.

Adjustment for either total walking or walking for transport did not materially alter the principal non-linear environmental indicator–CMB health associations. The non-linear associations of NO_2_ and PM_2.5_ with systolic blood pressure and of major road density and distance to the nearest busy road with HbA1c remained evident after adjustment for the respective walking measures (Figures S7.1 and S7.2). None of these associations formed part of a combination of component associations meeting the joint-significance criterion.

Spatial scale sensitivity analyses using 200 m and 500 m road-density measures and corresponding covariate specifications showed both similarities and some scale-specific differences in the walking associations (Tables S7.3 and S7.4). Total road density was inversely associated with total walking time among walkers under the 500 m and 1 km specifications, whereas major road density was inversely associated with this outcome only under the 1 km specification. In contrast, the positive association between distance to the nearest busy road and total walking time among walkers was consistent across spatial specifications. For walking for transport, total road density was positively associated with participation under the 200 m and 500 m specifications but not the primary 1 km specification. Positive associations of NO_2_ and PM_2.5_ with participation in walking for transport, and the inverse association of distance to the nearest busy road with participation, were consistent across all three spatial specifications.

## 4. Discussion

This study examined associations of ambient air pollution and road-related characteristics with multiple cardiometabolic health indicators in middle-aged and older adults living in Australian cities, while also investigating whether patterns involving total walking and walking for transport were consistent with hypothesized behavioral pathways. Several findings are noteworthy. First, after adjustment for multiple testing, associations of the ambient air-pollution indicators with CMB health were concentrated primarily on blood pressure, with PM_2.5_ showing the more consistently adverse pattern. Second, road-related environmental indicators were associated principally with lipid and glycemic outcomes, although the strength and statistical support for some road-density associations varied across spatial specifications. Third, the walking analyses revealed different patterns for total walking and walking for transport. Higher road densities were associated principally with less total walking, whereas higher NO_2_ and PM_2.5_ concentrations were associated with a greater likelihood of walking for transport. These patterns highlight the importance of distinguishing ambient air-pollution indicators from road-related environmental characteristics and of considering different walking behaviors separately when interpreting relationships between urban environments and health.

### 4.1. Ambient Air Pollution and Cardiometabolic Health

The associations observed for PM_2.5_ were concentrated on blood pressure. Higher PM_2.5_ was associated with higher diastolic blood pressure, while its association with systolic blood pressure was non-linear. Both findings were supported across the primary and sensitivity spatial specifications, suggesting that they were relatively robust to the spatial definition of the neighborhood covariates. These findings are broadly consistent with previous epidemiological evidence linking long-term particulate-matter exposure with hypertension and other cardiometabolic outcomes [8,12,13,14,15,42,43,44,45,46,47,48,49,50]. Several biological mechanisms may contribute to these associations, including pulmonary and systemic inflammation, oxidative stress, endothelial dysfunction and autonomic imbalance [8,48,49,51,52]. Because ambient PM_2.5_ arises from multiple primary and secondary sources, these findings should be interpreted as associations with ambient particulate air pollution rather than specifically with traffic-related air pollution [16].

The NO_2_ findings were more complex. NO_2_ showed a non-linear association with systolic blood pressure that was supported across spatial specifications, whereas an inverse association with diastolic blood pressure was more evident in the 200 m and 500 m spatial specifications. The inverse association contrasts with the predominantly adverse associations reported in systematic reviews and large cohort studies [14,43,44,53]. These findings should therefore be interpreted cautiously. At the relatively low NO_2_ concentrations observed in Australian cities, the observed associations may reflect residual spatial confounding, exposure measurement error, correlations between NO_2_ and other features of the urban environment, or uncertainty in the estimated exposure–response relationship. Although experimental evidence suggests that short-term NO_2_ exposure may under some circumstances influence vascular function and blood pressure [54,55], the present findings do not provide sufficient evidence that NO_2_ has a protective effect. Longitudinal studies with more detailed characterization of traffic-related pollutant mixtures and other correlated environmental exposures are needed to determine whether these associations are reproducible.

### 4.2. Road-Related Environmental Characteristics and Cardiometabolic Health

The road-related environmental indicators were associated primarily with lipid and glycemic outcomes. Associations of total road density with lower HDL cholesterol and higher triglycerides, and of major road density with higher triglycerides, were supported in the primary 1 km analyses and remained evident at 500 m, but not at 200 m. This pattern suggests that these lipid associations may be more readily captured by road-network characteristics operating over a broader residential area than by the immediate environment surrounding the home. Previous studies have reported associations of long-term air pollution and traffic-related environmental exposures with adverse lipid profiles, including elevated triglycerides and reduced HDL cholesterol [10,11,15]. Potential mechanisms include systemic inflammation, oxidative stress and autonomic and metabolic dysfunction [8,48,51,52].

Road density, however, is not a direct measure of exposure to traffic-related air pollution. It may capture a combination of traffic intensity, street-network configuration, traffic noise and other characteristics of urban environments, while the relative contribution of these factors cannot be distinguished in the present study. Consequently, mechanisms proposed for traffic-related pollution cannot be directly attributed to the observed road-density associations.

The glycemic findings showed a somewhat different spatial pattern. The non-linear association between major road density and HbA1c was supported only for the primary 1 km measure, indicating that this finding was sensitive to the spatial scale at which major road density was defined. Interpretation of the shape of this association should be cautious, particularly at the upper end of major road density where observations were relatively sparse. In contrast, the non-linear association between distance to the nearest busy road and HbA1c was supported across all three spatial specifications. Unlike road density, this measure characterizes residential proximity to a potentially important traffic source but provides no information about traffic volume, vehicle composition or pollutant concentrations. Thus, its association with HbA1c likewise cannot be attributed specifically to traffic-related air pollution.

Taken together, these findings indicate that road-related environmental characteristics provide information about CMB health that is complementary to residential PM_2.5_ and NO_2_ concentrations, while also showing that some associations depend on how the residential road environment is spatially characterized. These measures should therefore be regarded as indicators of the broader road and traffic environment rather than as surrogate measures of a specific air-pollution exposure. Their relationships with walking behaviors provide an additional dimension to their interpretation and are considered in the following section.

### 4.3. Patterns Involving Walking Behaviors

The analyses involving walking revealed distinct patterns for total walking and walking for transport, underscoring the importance of considering walking purpose and amount separately. In the primary analyses, higher total and major road densities were associated with less total walking time among those who walked. In contrast, greater distance from the nearest busy road was associated with more total walking time. Neither PM_2.5_ nor NO_2_ was significantly associated with total walking time, and none of the environmental indicators was significantly associated with participation in total walking after FDR adjustment.

These associations with total walking were compatible with some of the hypothesized behavioral relationships considered in this study. Participation in total walking was associated with smaller waist circumference and lower HbA1c, while greater total walking time was associated with smaller waist circumference, higher HDL cholesterol and lower triglycerides. When considered together with the environmental indicator–walking associations, these findings suggest a pattern in which higher total and major road densities coincided with less total walking time among walkers and less favorable levels of waist circumference, HDL cholesterol and triglycerides. Conversely, greater distance from a busy road was associated with more total walking time, which was itself associated with more favorable levels of these outcomes. Both component associations in each of these patterns met the FDR-adjusted significance threshold. However, no corresponding patterns involving participation in total walking met the joint-significance criterion after FDR adjustment. These patterns are broadly consistent with evidence linking walking and other forms of physical activity with better cardiometabolic health [22,23,24]. An exception was the positive association between total walking time and LDL cholesterol, which produced patterns in the opposite direction for this outcome. This unexpected finding may reflect residual confounding, including by dietary or other behavioral factors that strongly influence LDL cholesterol [56,57], and warrants cautious interpretation.

Walking for transport showed a different environmental pattern. Higher residential NO_2_ and PM_2.5_ concentrations were associated with a greater likelihood of participating in walking for transport, whereas greater distance from the nearest busy road was associated with a lower likelihood of doing so. Neither total nor major road density was significantly associated with participation in walking for transport in the primary 1 km analyses, and none of the environmental indicators was associated with walking-for-transport frequency among those who engaged in the behavior. Participation in walking for transport was also associated with smaller waist circumference and higher HDL cholesterol. Taken together, these component associations yielded FDR-supported patterns in which higher NO_2_ and PM_2.5_ concentrations coincided with greater participation in walking for transport and more favorable levels of these two CMB indicators; the corresponding pattern for distance from a busy road was in the opposite direction.

The positive associations of NO_2_ and PM_2.5_ with participation in walking for transport should not be interpreted as indicating that air pollution encourages walking. Rather, ambient pollutant concentrations and opportunities for transport walking may spatially covary because both are related to characteristics of urban form and activity patterns. More compact, destination-rich urban environments can provide greater opportunities for walking to daily destinations [1,2,20,21] while also concentrating traffic, population and other sources of air pollution [1]. Although the analyses adjusted for population density, land use, street-intersection density, access to rail and other neighborhood characteristics, these measures cannot fully characterize destination accessibility or other aspects of urban form. Residual spatial confounding therefore remains a plausible explanation for the positive pollutant–transport-walking associations. Importantly, the findings do not imply that PM_2.5_ or NO_2_ should themselves be regarded as indicators of destination accessibility.

Taken together, the contrasting findings for total walking and walking for transport illustrate why environmental indicator–health relationships cannot readily be reduced to a single “walking pathway”. Total walking includes walking undertaken for different purposes, while walking for transport depends particularly on the availability and accessibility of destinations [20,21,58]. Road-related characteristics may therefore have different relationships with recreational, utilitarian and overall walking. More broadly, the observed coexistence of adverse environmental indicator–CMB associations and more favorable patterns involving walking for transport also illustrates the importance of considering environmental exposures and health-supportive behaviors simultaneously when evaluating urban environments. Similar considerations underpin health-impact assessments showing that active travel can yield substantial population-health benefits while also altering exposure to air pollution and other transport-related hazards [3,23,24].

The interpretation of the above association patterns as being consistent with potential behavioral pathways should also take account of the temporal and measurement characteristics of the walking measures. Walking was self-reported for the previous week and may therefore not fully represent participants’ habitual walking over the longer period relevant to environmental exposure and CMB health. Thus, although the observed associations are compatible with the hypothesized behavioral pathways, the extent to which past-week walking reflects the longer-term behavioral patterns underlying these relationships is uncertain.

Furthermore, these findings do not demonstrate that walking mediates associations between environmental indicators and CMB health. The joint-significance analyses identify combinations in which both the environmental indicator–walking and walking–CMB associations met the FDR-adjusted significance threshold, but the cross-sectional design cannot establish their temporal ordering. In particular, while residential environmental characteristics plausibly precede current walking behavior, the direction of associations between walking and CMB health cannot be established from these data. The observed patterns can therefore inform hypotheses about possible behavioral pathways, but longitudinal data with temporally ordered measures would be required to evaluate such pathways more directly.

### 4.4. Spatial Scale

The spatial scale sensitivity analyses provide further insight into these relationships. Most environmental indicator–cardiometabolic associations were broadly consistent across spatial specifications, but several walking associations varied according to buffer size. Total road density was inversely associated with total walking time among walkers under 500 m and 1 km specifications, while the association with major road density met the FDR-adjusted threshold only under the 1 km specification. Conversely, total road density was positively associated with participation in walking for transport under 200 m and 500 m specifications but not under the primary 1 km specification.

These differences reinforce the importance of the spatial scale at which neighborhood characteristics are defined. Previous studies have shown that associations between built-environment attributes and walking can vary with buffer size and shape [59], while work examining transport walking has also demonstrated that the geographic definition of neighborhood exposure can influence estimated associations [60]. The 1 km specification was selected a priori for the primary analyses because it approximates a plausible neighborhood walking range for middle-aged and older adults [34,35]. The sensitivity findings nevertheless suggest that environmental characteristics operating close to the home may be related differently to walking than characteristics of the wider neighborhood. They also argue against selecting a spatial scale retrospectively on the basis of which specification produces the strongest or statistically significant association.

### 4.5. Implications

The findings highlight the need to consider air quality, road environments and active mobility together when planning health-supportive cities. Urban environments can provide opportunities for walking while simultaneously exposing residents to ambient air pollution and other adverse characteristics associated with road traffic. The positive associations of NO_2_ and PM_2.5_ with participation in walking for transport illustrate this potential coexistence: areas in which residents are more likely to walk to destinations may also have higher ambient pollutant concentrations. Improving opportunities for active travel should therefore be accompanied by efforts to reduce pollutant emissions and minimize pedestrians’ exposure to motorized traffic.

These findings are consistent with planning approaches that improve access to everyday destinations while reducing dependence on and exposure to motor vehicles. Relevant strategies may include locating frequently used destinations within walking distance of residences, providing connected and attractive pedestrian networks, reducing traffic volumes and vehicle emissions, and, where feasible, separating pedestrian routes from heavily trafficked roads. Such approaches need not involve a trade-off between walkability and air quality; rather, the aim should be to create neighborhoods in which residents can obtain the health benefits of walking without unnecessarily increasing their exposure to air pollution and other traffic-related environmental hazards [3,24].

The findings also have implications for research and health-impact assessment. Evaluations of urban and transport interventions should consider both changes in health-supportive behaviors, such as walking, and changes in environmental exposures. Examining behavioral changes or environmental exposures in isolation may provide an incomplete account of the potential health consequences of an intervention. Moreover, ambient pollutant concentrations and road-related environmental indicators should be treated as complementary but conceptually distinct measures: the former characterize ambient air pollution, whereas the latter capture aspects of the residential road environment that may be related to several environmental exposures and behaviors. Using multiple environmental measures alongside behavioral outcomes may therefore provide a more informative assessment of how urban environments relate to population health.

### 4.6. Strengths and Limitations

This study has several strengths. It examined multiple ambient air-pollution and road-related environmental indicators together with a broad range of objectively assessed cardiometabolic outcomes in a large sample drawn from Australian cities. PM_2.5_ and NO_2_ concentrations were estimated using nationally developed and validated models [30,31], while the road-related measures were objectively derived using Geographic Information Systems (GIS). The analysis accounted for a comprehensive set of individual- and neighborhood-level covariates informed by a DAG, including residential self-selection, and GAMMs allowed potentially non-linear exposure–response relationships to be identified. The explicit designation of a primary spatial specification, accompanied by analyses at alternative spatial scales, also provides a clearer assessment of robustness than treating all buffer sizes as interchangeable. Benjamini–Hochberg FDR adjustment within conceptually related test families addressed multiplicity, and the joint-significance criterion required both component associations to meet the FDR-adjusted threshold.

Several limitations warrant consideration. Most importantly, the analyses were cross-sectional. Temporal ordering between walking and cardiometabolic health cannot therefore be established, and the patterns satisfying the joint-significance criterion should not be interpreted as demonstrating that walking causally mediated the environmental indicator–health associations. Cross-sectional mediation analyses can provide biased representations of processes that unfold longitudinally [61]. Although residential environmental characteristics are relatively stable and plausibly precede walking behavior, reverse causation between walking and cardiometabolic health remains possible.

Second, environmental exposures were assigned according to residential address and did not capture participants’ exposures across daily activity spaces or along walking and travel routes. This is particularly relevant when studying walking for transport, because pollutant exposure during travel may differ from residential ambient concentrations. Road density and distance to a busy road also do not quantify traffic volume or individual exposure to traffic-generated pollutants. Traffic noise, indoor air pollution and other correlated environmental exposures were not assessed. Interpretation of the shapes of the non-linear associations at the extremes of the exposure distributions also warrants caution, as relatively few observations contributed to some of these regions, resulting in greater uncertainty in the fitted exposure–response functions.

Third, walking was self-reported for the previous week, whereas the ambient air-pollution indicators represented longer-term average residential exposure. The use of a short, clearly defined recall period is common in physical-activity assessment because it facilitates recall and reduces the cognitive burden associated with estimating usual or habitual activity. Nevertheless, walking during a single week may not fully represent participants’ habitual walking over the longer period relevant to environmental exposure and CMB health. Self-reported walking is also subject to recall and reporting error. Measurement error in the walking measures may not be purely random and could affect the two model components (i.e., participation and amount/frequency among the participants reporting the behavior) differently, potentially attenuating, inflating, or otherwise distorting the estimated associations in ways that are difficult to predict. The two-part modeling approach accommodates the point mass at zero but does not correct recall or reporting error in either component. These temporal and measurement limitations constrain interpretation of the observed associations as reflecting longer-term behavioral relationships.

Fourth, despite extensive covariate adjustment, residual confounding by individual and environmental characteristics remains possible. Although Benjamini–Hochberg FDR adjustment was applied within conceptually related test families, the large number of environmental indicator–walking–health combinations examined means that chance findings remain possible. FDR control applies within the specified families rather than across all analyses collectively. Findings should therefore be considered in relation to their effect estimates and confidence intervals and the consistency of patterns across related outcomes and spatial specifications. Differences in whether associations met the FDR-adjusted threshold across spatial specifications should not, by themselves, be interpreted as evidence that the associations differed statistically between specifications. Finally, attrition between the baseline and third wave of AusDiab may have introduced selection bias and may limit generalizability to less healthy or less mobile populations.

Longitudinal studies with temporally separated measurements of environmental conditions, walking behaviors and cardiometabolic outcomes are needed to evaluate the proposed pathways more rigorously. Such studies would benefit from incorporating GPS-derived activity spaces and walking routes, objective physical-activity measures, purpose-specific walking, traffic volume and composition, traffic noise, and higher-resolution or personal air-pollution exposure measures.

## 5. Conclusions

Ambient air pollution and road-related characteristics showed distinct associations with cardiometabolic health and walking behaviors among middle-aged and older adults living in Australian cities. PM_2.5_ showed the most consistent adverse associations among the ambient air-pollution indicators, whereas NO_2_ displayed a more mixed pattern. Higher road densities were associated with adverse lipid profiles, and major road density showed a non-linear association with HbA1c, while their associations with walking varied by walking domain and spatial scale.

The walking analyses further showed that the same environmental indicator may be associated differently with total walking and walking for transport. Higher total and major road densities were associated with less total walking time among walkers in the primary analyses, whereas higher NO_2_ and PM_2.5_ concentrations were associated with greater participation in walking for transport. Several combinations of environmental indicator–walking and walking–cardiometabolic associations remained supported after FDR adjustment and were compatible with hypothesized behavioral relationships. However, given the cross-sectional design, these patterns do not establish causal mediation. More broadly, the findings emphasize the need to distinguish ambient air pollution from road-related environmental characteristics and to consider both environmental exposures and mobility behaviors when evaluating the health implications of urban environments.

## Figures and Tables

**Table 1:** Participant characteristics (N = 4141).

Characteristics	Statistics	Characteristics	Statistics
*Individual-level sociodemographic characteristics*			
Age, years, M ± SD	61.1 ± 11.4	Sex, female, %	55.2
Educational attainment, %		Employment status, %	
Up to secondary	32.7	Not employed	30.4
Trade, technician certificate	29.1	Paid employment	52.2
Associate diploma and equivalent	14.5	Volunteering	15.1
Bachelor’s degree, post-graduate diploma	23.1	Missing data	2.3
Missing data	0.6	English-speaking background, %	89.9
Living arrangements, %		Household income, annual, %	
Couple without children	48.2	Up to $49,999	32.9
Couple with children	26.8	$50,000–$99,999	26.8
Other	22.4	$100,000 and over	28.9
Missing data	2.4	Does not know or refusal	8.8
		Missing data	2.7
Residential self-selection—access to destinations, M ± SD	3.0 ± 1.4	Residential self-selection—recreational facilities, M ± SD	3.1 ± 1.5
Missing data, %	7.8	Missing data, %	7.8
***Walking, M ± SD, % missing data***			
Measure: Total walking, 0.0%		Measure: Walking for transport, 2.7%	
Total min/week, M ± SD	168.9 ± 195.8	Total times/week, M ± SD	1.4 ± 3.1
Any walking, %	80.6	Any walking for transport, %	29.9
Min/week in those reporting the behavior, M ± SD	209.7 ± 197.6	Times/week in those reporting the behavior, M ± SD	4.6 ± 5.1
***Cardiometabolic health indicators, M ± SD (outcomes), % missing data***			
Mean systolic BP, mmHg, 0.2%	130.1 ± 19.6	HbA1c, mmol/mol, 0.5%	39.9 ± 6.3
Mean diastolic BP, mmHg, 0.2%	72.9 ± 10.8	LDL cholesterol, mmol/L, 1.4%	3.0 ± 0.9
Waist circumference, cm, 0.2%	94.6 ± 14.2	Triglycerides, mmol/L, 0.3%	1.3 ± 0.9
HDL cholesterol, mmol/L, 0.3%	1.5 ± 0.4		
***Other health-related variables, %***			
Diabetes medication	6.3	Tobacco-smoking status	
Missing data	1.8	Current smoker	7.0
Antihypertensive medication	32.0	Previous smoker	35.9
Missing data	1.8	Non-smoker	54.5
Lipid-lowering medication	24.5	Missing data	2.6
Missing data	1.8	Heart problems/stroke history	8.7
		Missing data	1.0

Notes: M—mean; SD—standard deviation; HbA1c—glycated hemoglobin; LDL—low-density lipoprotein; HDL—high-density lipoprotein. BP—blood pressure.

**Table 2:** Descriptive statistics of environmental indicators and neighborhood-level covariates used in the main analyses (N = 4141).

Characteristic	Spatial Definition	M ± SD
*Ambient air-pollution indicator*		
PM_2.5_, μg/m^3^	Residential address	6.3 ± 1.7
NO_2_, ppb	Residential address	5.5 ± 2.1
*Road-related environmental indicators*		
Total road density, m/km^2^	1 km residential Euclidean buffer	9576 ± 3404
Major road density, m/km^2^	1 km residential Euclidean buffer	1314 ± 895
Distance to nearest busy road, m	Residential address	457 ± 498
*Neighborhood-level covariates*		
Population density, persons/ha	1 km residential Euclidean buffer	14.3 ± 9.1
Area-level IRSAD, in deciles	SA1 of residential address	6.4 ± 2.7
Parkland, %	1 km residential Euclidean buffer	14.7 ± 13.8
Blue space, %	1 km residential Euclidean buffer	0.3 ± 2.4
Commercial land use, %	1 km residential Euclidean buffer	2.5 ± 5.1
Other land uses, %	1 km residential Euclidean buffer	73.6 ± 18.8
Street intersection density, intersections/km^2^	1 km residential Euclidean buffer	46.4 ± 25.2
Distance to nearest train station, m	Residential address	7664 ± 8286

Notes: M—mean; SD—standard deviation; IRSAD—Index of Relative Socioeconomic Advantage and Disadvantage; NO_2_—nitrogen dioxide; PM_2.5_—particulate matter ≤ 2.5 μm aerodynamic diameter.

**Table 3:** Associations of ambient air pollution and road-related environmental indicators with cardiometabolic health outcomes (N = 4141).

CMB Health Outcome	Environmental Indicator	Estimate/Statistic	95% CI	*p*	*q*
Waist circumference	NO_2_ (ppb)	*b* = 0.057	−0.272, 0.385	0.734	0.734
(cm)	PM_2.5_ (μg/m^3^)	*b* = 0.359	0.053, 0.665	**0.022**	0.110
	Total road density (per 1000 m/km^2^; 1 km buffer)	*b* = 0.361	−0.033, 0.756	0.073	0.183
	Major road density (per 1000 m/km^2^; 1 km buffer)	*b* = 0.339	−0.246, 0.924	0.256	0.320
	Distance to nearest busy road (per 100 m)	*b* = −0.076	−0.177, 0.026	0.145	0.242
Systolic BP	NO_2_ (ppb)	**Non-linear ^1^**; Figure 1a	**—**	**0.002**	**0.005**
(mmHg)	PM_2.5_ (μg/m^3^)	**Non-linear ^1^**; Figure 1b	**—**	**<0.001**	**<0.001**
	Total road density (per 1000 m/km^2^; 1 km buffer)	*b* = −0.335	−0.849, 0.178	0.200	0.250
	Major road density (per 1000 m/km^2^; 1 km buffer)	*b* = −0.196	−0.958, 0.566	0.614	0.614
	Distance to nearest busy road (per 100 m)	*b* = 0.099	−0.032, 0.231	0.138	0.230
Diastolic BP (mmHg)	NO_2_ (ppb)	***b* = −0.261**	**−0.513, −0.009**	**0.042**	0.105
	PM_2.5_ (μg/m^3^)	***b* = 0.373**	**0.138, 0.609**	**0.002**	**0.010**
	Total road density (per 1000 m/km^2^; 1 km buffer)	*b* = −0.027	−0.334, 0.280	0.862	0.862
	Major road density (per 1000 m/km^2^; 1 km buffer)	*b* = 0.066	−0.388, 0.519	0.777	0.862
	Distance to nearest busy road (per 100 m)	*b* = 0.009	−0.069, 0.087	0.821	0.862
HDL-C	NO_2_ (ppb)	*b* = −0.004	−0.014, 0.005	0.376	0.470
(mmol/L)	PM_2.5_ (μg/m^3^)	*b* = −0.007	−0.016, 0.002	0.112	0.280
	Total road density (per 1000 m/km^2^; 1 km buffer)	***b* = −0.019**	**−0.031, −0.007**	**0.001**	**0.005**
	Major road density (per 1000 m/km^2^; 1 km buffer)	*b* = −0.009	−0.026, 0.008	0.311	0.470
	Distance to nearest busy road (per 100 m)	*b* = 0.001	−0.002, 0.004	0.686	0.686
LDL-C	NO_2_ (ppb)	*b* = −0.003	−0.023, 0.017	0.761	0.809
(mmol/L)	PM_2.5_ (μg/m^3^)	***b* = 0.020**	**0.001, 0.038**	**0.034**	0.170
	Total road density (per 1000 m/km^2^; 1 km buffer)	*b* = −0.003	−0.027, 0.021	0.809	0.809
	Major road density (per 1000 m/km^2^; 1 km buffer)	*b* = 0.017	−0.019, 0.053	0.348	0.809
	Distance to nearest busy road (per 100 m)	*b* = −0.002	−0.008, 0.004	0.544	0.809
Triglycerides	NO_2_ (ppb)	e*ᵇ* = 1.000	0.986, 1.013	0.973	0.973
(mmol/L)	PM_2.5_ (μg/m^3^)	e*ᵇ* = 1.006	0.993, 1.018	0.363	0.453
	Total road density (per 1000 m/km^2^; 1 km buffer)	**e*ᵇ* = 1.032**	**1.015, 1.049**	**<0.001**	**<0.001**
	Major road density (per 1000 m/km^2^; 1 km buffer)	**e*ᵇ* = 1.042**	**1.017, 1.067**	**<0.001**	**<0.001**
	Distance to nearest busy road (per 100 m)	e*ᵇ* = 0.998	0.994, 1.002	0.267	0.445
HbA1c	NO_2_ (ppb)	e*ᵇ* = 1.002	0.998, 1.006	0.349	0.582
(mmol/mol)	PM_2.5_ (μg/m^3^)	e*ᵇ* = 1.001	0.997, 1.005	0.636	0.636
	Total road density (per 1000 m/km^2^; 1 km buffer)	e*ᵇ* = 1.002	0.997, 1.006	0.499	0.624
	Major road density (per 1000 m/km^2^; 1 km buffer)	**Non-linear ^1^**; Figure 1c	**—**	**0.001**	**0.003**
	Distance to nearest busy road (per 100 m)	**Non-linear ^1^**; Figure 1d	**—**	**<0.001**	**0.001**

Notes: Road-density estimates refer to measures calculated within 1 km residential buffers. PM_2.5_ and NO_2_ were estimated at participants’ residential addresses, and aerial distance to the nearest busy road was calculated from the residential address; these three indicators were not defined using residential buffers. GAMMs with Gaussian variance and identity link functions were used for waist circumference, systolic and diastolic BP, HDL-C and LDL-C. GAMMs with Gamma variance and logarithmic link functions were used for triglycerides and HbA1c. All models were adjusted for the covariates specified in Table S3.1. *b*—regression coefficient; e*ᵇ*—exponentiated regression coefficient; CI—confidence interval; BP—blood pressure; HDL-C—high-density lipoprotein cholesterol; LDL-C—low-density lipoprotein cholesterol; HbA1c—glycated hemoglobin; NO_2_—nitrogen dioxide; PM_2.5_—particulate matter with an aerodynamic diameter ≤ 2.5 μm; *p*—*p*-value; *q*—Benjamini–Hochberg false discovery rate (FDR)-adjusted *p*-value. Bold estimates/statistics, CIs and *p*-values indicate *p* < 0.05 and bold *q*-values indicate *q* < 0.05. ^1^ Pooled *p*-value for the smooth term obtained using a D1 multivariate Wald test based on Rubin’s multiple-imputation combining rules.

## Data Availability

Modeled data are reported in this manuscript and in the supplementary materials. Raw data are available on request.

## Supplementary Materials

The following supporting information can be downloaded at https://www.mdpi.com/article/10.3390/toxics14100920/s1. S1. Distribution of AusDiab participants in Australia: Figure S1.1. Map showing the indicative location of AusDiab participants used in the study aged over 25 years. S2. DAG for environmental indicators and CMB health outcomes: Table S2.1. Description of DAG variables; Figure S2.1. Directed acyclic graph (DAG) showing simplified associations between environmental indicators and cardiometabolic health outcomes. S3. Outline of regression analyses: Table S3.1. Description of regression models examining associations of ambient air pollution and road-related environmental indicators with CMB health and association patterns involving walking. S4. Descriptive statistics of environmental indicators and neighborhood-level covariates used in the sensitivity analyses: Table S4.1. Descriptive statistics of environmental indicators and neighborhood-level covariates used in the sensitivity analyses (N = 4141). S5. Correlations among ambient air pollution and road-related environmental indicators: Table S5.1. Correlation among ambient air pollution and road-related environmental indicators (Spearman correlation and Pearson correlation coefficients in brackets) [primary analyses]; Table S5.2. Correlation among total and major road densities within 200 m Euclidean residential buffers, ambient air-pollution indicators and distance to busy road (Spearman correlation and Pearson correlation coefficients in brackets); Table S5.3. Correlation among total and major road densities within 500 m Euclidean residential buffers, ambient air-pollution indicators and distance to busy road (Spearman correlation and Pearson correlation coefficients in brackets). S6. Associations of ambient air pollution and road-related environmental indicators with cardiometabolic health outcomes (sensitivity analyses): Table S6.1. Sensitivity analyses of associations of ambient air pollution and road-related environmental indicators with cardiometabolic health outcomes: 200 m residential buffer specification; Table S6.2. Sensitivity analyses of associations of ambient air pollution and road-related environmental indicators with cardiometabolic health outcomes: 500 m residential buffer specification; Figure S6.1. Estimated non-linear association of major road density (500 m buffer) with systolic blood pressure. S7. Associations involving walking used to evaluate hypothesized behavioral relationships between environmental indicators and cardiometabolic health: Table S7.1. Primary analyses of associations involving total walking used to evaluate hypothesized behavioral relationships between environmental indicators and cardiometabolic health (1 km spatial specification); Figure S7.1. Total walking-adjusted non-linear associations of (a) NO_2_ with systolic blood pressure, (b) PM_2.5_ with systolic blood pressure, (c) major road density with HbA1c, and (d) aerial distance to the nearest busy road with HbA1c; Table S7.2. Primary analyses of associations involving walking for transport used to evaluate hypothesized behavioral relationships between environmental indicators and cardiometabolic health (1 km spatial specification); Figure S7.2. Walking for transport-adjusted non-linear associations of (a) NO_2_ with systolic blood pressure, (b) PM_2.5_ with systolic blood pressure, (c) major road density with HbA1c, and (d) aerial distance to the nearest busy road with HbA1c; Table S7.3. Spatial-scale sensitivity analyses of associations involving total walking used to evaluate hypothesized behavioral relationships between environmental indicators and cardiometabolic health; Figure S7.3. Total walking-adjusted estimated non-linear associations of major road density (500 m buffer) with systolic blood pressure; Table S7.4. Spatial-scale sensitivity analyses of associations involving walking for transport used to evaluate hypothesized behavioral relationships between environmental indicators and cardiometabolic health; Figure S7.4. Walking for transport-adjusted non-linear associations of major road density (500 m buffer) with systolic blood pressure.

## Abbreviations

The following abbreviations are used in this manuscript:

AusDiab	Australian Diabetes, Obesity and Lifestyle
BP	Blood pressure
CI	Confidence interval
CMB	Cardiometabolic
DAG	Directed acyclic graph
FDR	False discovery rate
GAMM	Generalized additive mixed model
HbA1c	Glycated hemoglobin
HDL	High-density lipoprotein
IRSAD	Index of relative socioeconomic advantage and disadvantage
km	kilometer
LDL	Low-density lipoprotein
M	Mean
m	meter
μ	micro
NO_2_	Nitrogen dioxide
OR	Odds ratio
PM_2.5_	Particulate matter with an aerodynamic diameter of ≤2.5 μm
ppb	parts per billion
RMSE	Root mean square error
SD	Standard deviation
TRAP	Traffic-related air pollution
