# Supplementary material for: Associations of Ambient Air Pollution and Road-Related Environmental Characteristics with Cardiometabolic Health: The Role of Walking
Source: Toxics. 2026 Oct 17;14(10):920. doi: 10.3390/toxics14100920 (PMC13709772; doi:10.3390/toxics14100920)
Supplement: Supplementary file 1 [file toxics-14-00920-s001.zip › toxics-4612456-supplementary.pdf]

Supplementary material

S1.1 Distribution of AusDiab participants in Australia

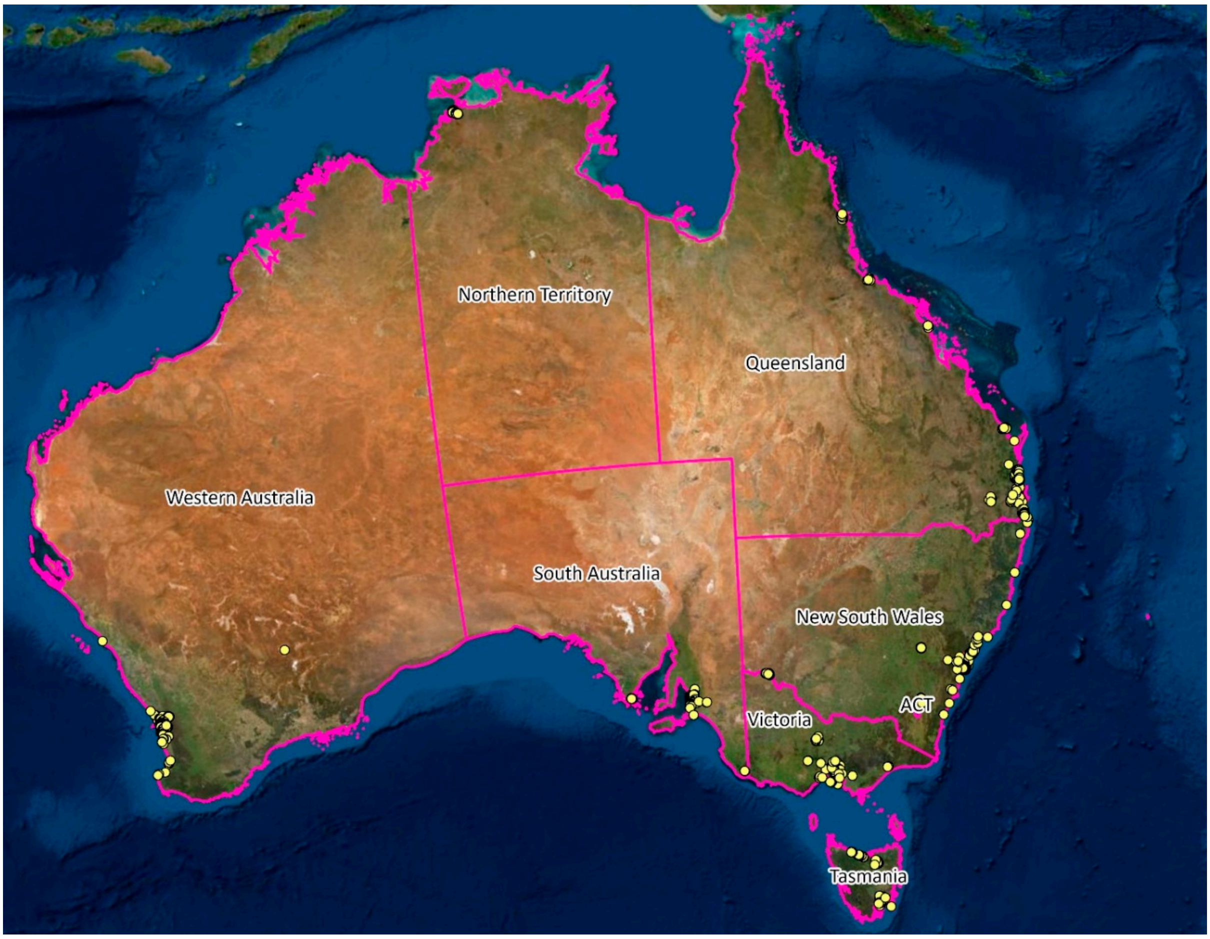

Figure S1.1 Map showing indicative location of AusDiab participants used in the study aged over 25 years.

S.2. DAG for environmental indicators and CMB health outcomes

Table S.2.1. Description of DAG variables.

DAG Variable	Description
Environmental indicators	• NO_2<br>• $PM_{2.5}$<br>• Total road density<br>• Major road density<br>• Distance to busy road
Area SES	Area-level socioeconomic status, IRSAD
Individual SES	Educational attainment
Hh income	Household income
Eng sb	English speaking background
Sex	Male/Female
NSS_rec	Neighborhood self-selection – recreational facilities
NSS dest	Neighborhood self-selection – access to destinations
Living arrangement	Living arrangements
Work status	Employment status
CMB health (outcomes)	• Waist circumference<br>• Systolic BP<br>• Diastolic BP<br>• HDL<br>• LDL<br>• Triglycerides<br>HbA1c
Age	Age
SI density	Street intersection density
Land use	ABS meshblock land use classification:<br>Commercial<br>Industrial<br>Mix of other land uses (Medical, Education, Residential, Agricultural, Blue space, Other)
Train station dt	Distance to nearest train station
Parkland	Percentage of parkland within buffer
Pp density	Population density
Smoking	Tobacco-smoking status
Hx CVD	Previous cardiovascular disease history

Notes: DAG, Directed acyclic graph; LDL, low-density lipoprotein; HDL, high-density lipoprotein; HbA1c, glycated hemoglobin; IRSAD, Index of Relative Socioeconomic Advantage and Disadvantage; NO_2, nitrogen dioxide; $PM_{2.5}$, particulate matter ≤ 2.5 μm aerodynamic diameter; ABS, Australian Bureau of Statistics; CMB, cardiometabolic; BP, blood pressure.

We identified 17 variables as potential confounders of associations or predictors of CMB health outcomes. The variables were: area-level socioeconomic status (SES) (Index of Relative Socioeconomic Advantage and Disadvantage - IRSAD), individual-level educational attainment (up to secondary education; trade, technician certificate; associate diploma or equivalent; Bachelor degree, post-graduate diploma), annual household income (up to $49,999; $50,000 – $99,999; $100,000 and over; does not know or refused to answer), English-speaking background (Yes/No), neighborhood (residential) self-selection (NSS) in relation to destinations of daily living and active leisure activities, work status (paid work; volunteer: or neither), living arrangements (couple living with children;

couple living without children; other types of living arrangements), age, sex, tobacco smoking status (never smoker or smoker), and history of CVDs (heart problems or stroke). In addition, population density (number of people per hectare of residential buffers) street intersection density (total count of ≥ 3-way road intersections per km^2 within the residential buffers), land use (proportion/percentage of land use area type[s] within the residential buffers), aerial distance to nearest train stop and railway were also included as they relate to road and traffic neighborhood characteristics that may influence a person's walking behaviors and exposure to ambient air-pollution and road-related environmental characteristics.

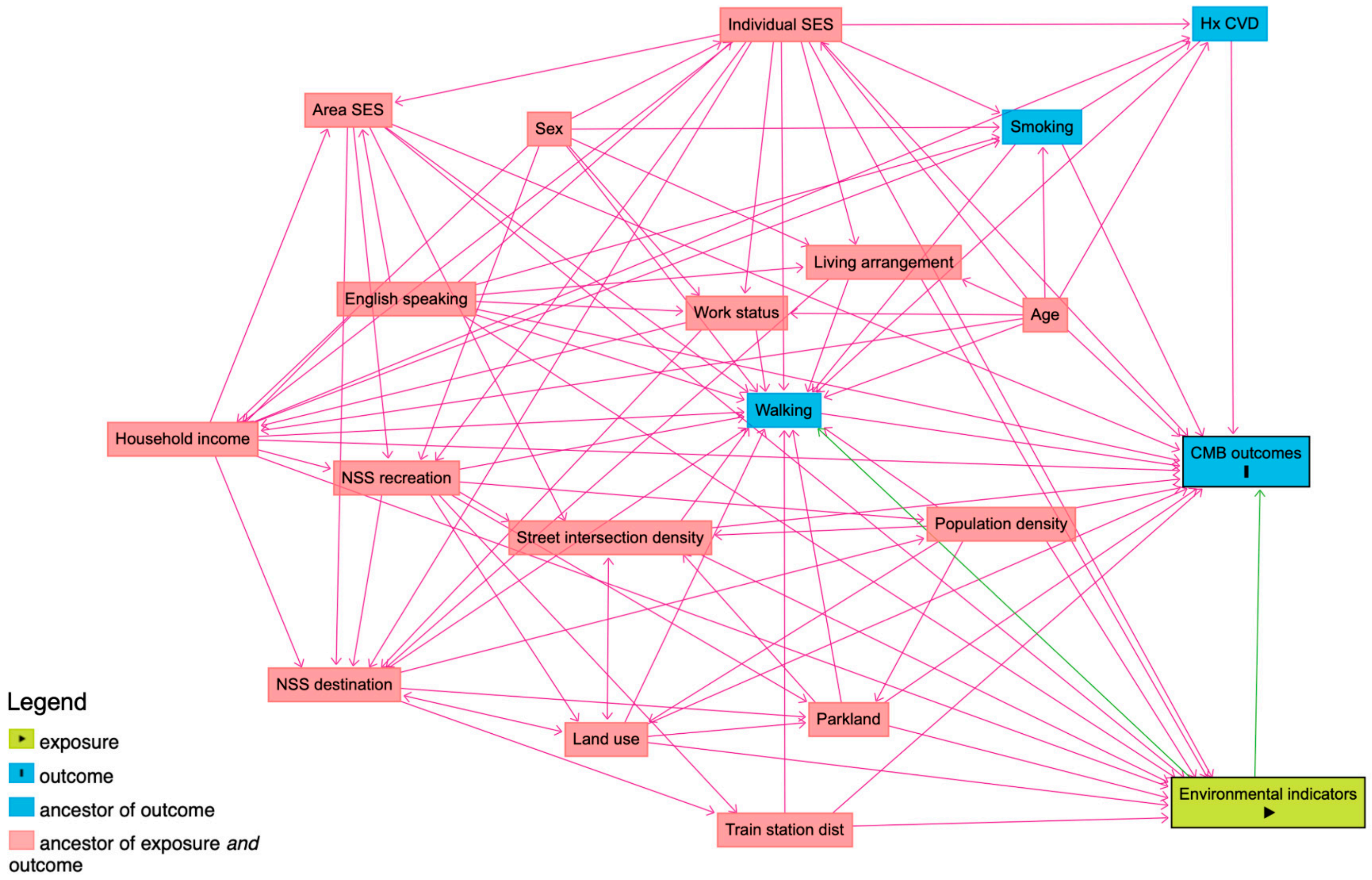

Figure S.2.1. Directed acyclic graph (DAG) showing simplified associations between environmental indicators and cardiometabolic health outcomes.

S.3. Outline of regression analyses

Table S.3.1. Description of regression models examining associations of ambient air pollution and road-related environmental indicators with CMB health and association patterns involving walking.

Analysis component	Outcome	Environmental indicator(s) / walking variable	Confounders/covariates	Regression model	Results table/figure
**Environmental indicators & CMB health**	Waist circumference, systolic BP, diastolic BP, HDL-C, LDL-C	$PM_{2.5}$, NO_2, total road density, major road density, distance to nearest busy road	Area- and individual-level SES; household income; English-speaking background; sex; residential self-selection for recreation and destination accessibility; living arrangement; work status; age; street intersection density; land use; distance to nearest train station; parkland; population density; smoking status; history of cardiovascular disease	GAMMs with Gaussian variance and identity link. Environmental indicators modelled using linear or smooth terms, as appropriate.	Tables 3/S.6.1/S.6.2
**Environmental indicators & CMB health**	Triglycerides, HbA1c	$PM_{2.5}$, NO_2, total road density, major road density, distance to nearest busy road	Area- and individual-level SES; household income; English-speaking background; sex; residential self-selection for recreation and destination accessibility; living arrangement; work status; age; street intersection density; land use; distance to nearest train station; parkland; population density; smoking status; history of cardiovascular disease	GAMMs with Gamma variance and logarithmic link. Environmental indicators modelled using linear or smooth terms, as appropriate.	Tables 3/S.6.1/S.6.2
**Environmental indicators & total walking**	Participation in total walking in the previous week (yes/no)	$PM_{2.5}$, NO_2, total road density, major road density, aerial distance to nearest busy road	Area- and individual-level SES; household income; English-speaking background; sex; residential self-selection for recreation and destination accessibility; living arrangement; work status; age; street intersection density; land use; distance to nearest train station; parkland; population density; smoking status; history of cardiovascular disease	GAMMs with binomial variance and logit link. Environmental indicators modelled using linear or smooth terms, as appropriate.	Tables S.7.1/S.7.3

Analysis component	Outcome	Environmental indicator(s) / walking variable	Confounders/covariates	Regression model	Results table/figure
**Environmental indicators & total walking**	Non-zero total walking time (min/week), among participants reporting any walking	$PM_{2.5}$, NO_2, total road density, major road density, distance to nearest busy road	Area- and individual-level SES; household income; English-speaking background; sex; residential self-selection for recreation and destination accessibility; living arrangement; work status; age; street intersection density; land use; distance to nearest train station; parkland; population density; smoking status; history of cardiovascular disease	GAMMs with Gamma variance and logarithmic link. Environmental indicators modelled using linear or smooth terms, as appropriate.	Tables S.7.1/S.7.3
**Environmental indicators & walking for transport**	Participation in walking for transport in the previous week (yes/no)	$PM_{2.5}$, NO_2, total road density, major road density, distance to nearest busy road	Area- and individual-level SES; household income; English-speaking background; sex; residential self-selection for recreation and destination accessibility; living arrangement; work status; age; street intersection density; land use; distance to nearest train station; parkland; population density; smoking status; history of cardiovascular disease	GAMMs with binomial variance and logit link. Environmental indicators modelled using linear or smooth terms, as appropriate.	Tables S.7.2/S.7.4
**Environmental indicators & walking for transport**	Non-zero frequency of walking for transport (times/week), among participants reporting any walking for transport	$PM_{2.5}$, NO_2, total road density, major road density, distance to nearest busy road	Area- and individual-level SES; household income; English-speaking background; sex; residential self-selection for recreation and destination accessibility; living arrangement; work status; age; street intersection density; land use; distance to nearest train station; parkland; population density; smoking status; history of cardiovascular disease	GAMMs with negative-binomial variance and logarithmic link. Environmental indicators modelled using linear or smooth terms, as appropriate.	Tables S.7.2/S.7.4
**Total walking & CMB health, adjusted for environmental indicator**	Waist circumference, systolic BP, diastolic BP, HDL-C, LDL-C	Participation in total walking and/or non-zero total walking time; relevant environmental	Area- and individual-level SES; household income; English-speaking background; sex; residential self-selection for recreation and destination accessibility; living arrangement; work	GAMMs with Gaussian variance and identity link, including the relevant total-walking variable and	Tables S.7.1/S.7.3

Analysis component	Outcome	Environmental indicator(s) / walking variable	Confounders/covariates	Regression model	Results table/figure
		indicator included in the model	status; age; street intersection density; land use; distance to nearest train station; parkland; population density; smoking status; history of cardiovascular disease; relevant environmental indicator	environmental indicator.	
**Total walking & CMB health, adjusted for environmental indicator**	Triglycerides, HbA1c	Participation in total walking and/or non-zero total walking time; relevant environmental indicator included in the model	Area- and individual-level SES; household income; English-speaking background; sex; residential self-selection for recreation and destination accessibility; living arrangement; work status; age; street intersection density; land use; distance to nearest train station; parkland; population density; smoking status; history of cardiovascular disease; relevant environmental indicator	GAMMs with Gamma variance and logarithmic link, including the relevant total-walking variable and environmental indicator.	Tables S.7.1/S.7.3
**Walking for transport & CMB health, adjusted for environmental indicator**	Waist circumference, systolic BP, diastolic BP, HDL-C, LDL-C	Participation in walking for transport and/or non-zero frequency of walking for transport; relevant environmental indicator included in the model	Area- and individual-level SES; household income; English-speaking background; sex; residential self-selection for recreation and destination accessibility; living arrangement; work status; age; street intersection density; land use; distance to nearest train station; parkland; population density; smoking status; history of cardiovascular disease; relevant environmental indicator	GAMMs with Gaussian variance and identity link, including the relevant walking-for-transport variable and environmental indicator.	Tables S.7.2/S.7.4
**Walking for transport & CMB health, adjusted for environmental indicator**	Triglycerides, HbA1c	Participation in walking for transport and/or non-zero frequency of walking for transport; relevant environmental	Area- and individual-level SES; household income; English-speaking background; sex; residential self-selection for recreation and destination accessibility; living arrangement; work status; age; street intersection density; land use; distance to nearest train station;	GAMMs with Gamma variance and logarithmic link, including the relevant walking-for-transport variable and	Tables S.7.2/S.7.4

Analysis component	Outcome	Environmental indicator(s) / walking variable	Confounders/covariates	Regression model	Results table/figure
		indicator included in the model	parkland; population density; smoking status; history of cardiovascular disease; relevant environmental indicator	environmental indicator.	

Notes: Covariate adjustment was informed by directed acyclic graphs (DAGs). Cross-sectional association patterns involving walking were evaluated using the joint-significance criterion, requiring statistical support for both an association between the environmental indicator and the relevant walking measure and an association between that walking measure and the CMB health outcome after adjustment for the environmental indicator. Given the cross-sectional design, these analyses were interpreted as patterns of associations consistent with hypothesized behavioral pathways rather than as evidence of temporal ordering, indirect effects or causal mediation. Walking outcomes were analyzed using a two-part modelling strategy because of the large proportion of zero values. Participation in total walking and walking for transport was modelled using binomial GAMMs with logit links. Among participants reporting the respective behavior, non-zero total walking time was modelled using a Gamma distribution with a logarithmic link and non-zero frequency of walking for transport using a negative-binomial distribution with a logarithmic link. The functional form of associations was evaluated by comparing Akaike Information Criterion (AIC) values for models containing linear and smooth terms. Evidence supporting a non-linear association was considered present when the model containing the smooth term had an AIC at least 10 units lower than the corresponding linear model. All other model specifications were identical between the models being compared. CMB, cardiometabolic; $PM_{2.5}$, particulate matter with an aerodynamic diameter ≤2.5 μm; NO_2, nitrogen dioxide; SES, socioeconomic status; GAMM, generalized additive mixed model; BP, blood pressure; HDL-C, high-density lipoprotein cholesterol; LDL-C, low-density lipoprotein cholesterol; HbA1c, glycated hemoglobin.

S.4. Descriptive statistics of environmental indicators and neighborhood-level covariates used in the sensitivity analyses

Table S.4.1. Descriptive statistics of environmental indicators and neighborhood-level covariates used in the sensitivity analyses (N = 4,141).

Characteristic	Spatial definition	M ± SD
Road-related environmental indicators		
Total road density, m/km^2	200-m residential Euclidean buffer	11,713 ± 4,197
	500-m residential Euclidean buffer	10,742 ± 3,766
Major road density, m/km^2	200-m residential Euclidean buffer	965 ± 1,684
	500-m residential Euclidean buffer	1,362 ± 1,329
Neighborhood-level covariates		
Population density, persons/ha	200-m residential Euclidean buffer	20.3 ± 12.2
	500-m residential Euclidean buffer	16.9 ± 10.2
Parkland, %	200-m residential Euclidean buffer	7.6 ± 12.5
	500-m residential Euclidean buffer	11.7 ± 13.8
Blue space, %	200-m residential Euclidean buffer	0.2 ± 2.3
	500-m residential Euclidean buffer	0.2 ± 2.5
Commercial land use, %	200-m residential Euclidean buffer	1.3 ± 6.6
	500-m residential Euclidean buffer	2.1 ± 5.9
Other land uses, %	200-m residential Euclidean buffer	89.0 ± 15.9
	500-m residential Euclidean buffer	80.8 ± 18.2
Street intersection density, intersections/km^2	200-m residential Euclidean buffer	60.9 ± 39.4
	500-m residential Euclidean buffer	53.8 ± 30.5

Note: The ambient air-pollution indicators ($PM_{2.5}$ and NO_2) used in the sensitivity analyses were the same as those used in the primary analyses because their spatial definition was based on the residential address rather than a residential buffer of a specific size. M, mean; SD, standard deviation.

S.5. Correlations among ambient air-pollution and road-related environmental indicators

Table S.5.1. Correlation among ambient air-pollution and road-related environmental indicators (Spearman correlation and Pearson correlation coefficients in brackets) [primary analyses].

Indicators	NO_2	$PM_{2.5}$	Total road density (1-km)	Major road density (1-km)	Distance to busy road
NO_2	1.00	0.27 (0.25)	0.39 (0.43)	0.40 (0.38)	-0.35 (-0.24)
$PM_{2.5}$	0.27 (0.25)	1.00	0.38 (0.39)	0.30 (0.29)	-0.23 (-0.29)
Total road density (1-km)	0.39 (0.43)	0.38 (0.39)	1.00	0.44 (0.47)	-0.25 (-0.34)
Major road density (1-km)	0.40 (0.38)	0.30 (0.29)	0.44 (0.47)	1.00	**-0.63 (-0.60)**
Distance to busy road	-0.35 (-0.24)	-0.23 (-0.29)	-0.25 (-0.34)	**-0.63 (-0.60)**	1.00

Notes: NO_2, nitrogen dioxide; $PM_{2.5}$, particulate matter with aerodynamic diameter ≤ 2.5 μm.

Table S.5.2. Correlation among total and major road densities within 200-m Euclidean residential buffers, ambient air-pollution indicators and distance to busy road (Spearman correlation and Pearson correlation coefficients in brackets).

Indicators	NO_2	$PM_{2.5}$	Total road density (200-m)	Major road density (200-m)	Distance to busy road
NO_2	1.00	0.27 (0.25)	0.21 (0.25)	0.25 (0.23)	-0.35 (-0.24)
$PM_{2.5}$	0.27 (0.25)	1.00	0.25 (0.25)	0.17 (0.13)	-0.23 (-0.29)
Total road density (200-m)	0.21 (0.25)	0.25 (0.25)	1.00	0.27 (0.29)	-0.22 (-0.25)
Major road density (200-m)	0.25 (0.23)	0.17 (0.13)	0.27 (0.29)	1.00	**-0.76 (-0.43)**
Distance to busy road	-0.35 (-0.24)	-0.23 (-0.29)	-0.22 (-0.25)	**-0.76 (-0.43)**	1.00

Notes: NO_2, nitrogen dioxide; $PM_{2.5}$, particulate matter with aerodynamic diameter ≤ 2.5 μm.

Table S.5.3. Correlation among total and major road densities within 500-m Euclidean residential buffers, ambient air-pollution indicators and distance to busy road (Spearman correlation and Pearson correlation coefficients in brackets).

Indicators	NO_2	$PM_{2.5}$	Total road density (500-m)	Major road density (500-m)	Distance to busy road
NO_2	1.00	0.27 (0.25)	0.34 (0.38)	0.43 (0.41)	-0.35 (-0.24)
$PM_{2.5}$	0.27 (0.25)	1.00	0.35 (0.35)	0.19 (0.16)	-0.23 (-0.29)
Total road density (500-m)	0.34 (0.38)	0.35 (0.35)	1.00	0.35 (0.41)	-0.29 (-0.35)
Major road density (500-m)	0.43 (0.41)	0.19 (0.16)	0.35 (0.41)	1.00	**-0.85 (-0.59)**
Distance to busy road	-0.35 (-0.24)	-0.23 (-0.29)	-0.29 (-0.35)	**-0.85 (-0.59)**	1.00

Notes: NO_2, nitrogen dioxide; $PM_{2.5}$, particulate matter with aerodynamic diameter ≤ 2.5 μm.

S.6. Associations of ambient air pollution and road-related environmental indicators with cardiometabolic health outcomes (sensitivity analyses).

Table S.6.1. Sensitivity analyses of associations of ambient air pollution and road-related environmental indicators with cardiometabolic health outcomes: 200-m residential buffer specification.

CMB health outcome	Environmental indicator	Estimate/statistic	95% CI	*p*	*q*
Waist circumference (cm)	NO_2 (ppb)	*b* = 0.048	−0.243, 0.338	0.748	0.771
	$PM_{2.5}$ (μg/m³)	***b* = 0.328**	**0.037, 0.619**	**0.027**	0.135
	Total road density (per 1000 m/km²; 200-m buffer)	*b* = 0.085	−0.128, 0.298	0.432	0.720
	Major road density (per 1000 m/km²; 200-m buffer)	*b* = −0.039	−0.305, 0.226	0.771	0.771
	Distance to nearest busy road (per 100 m)	*b* = −0.071	−0.170, 0.028	0.159	0.398
Systolic BP (mmHg)	NO_2 (ppb)	**Non-linear[1]; Fig. 1a**	—	**<0.001**	**<0.001**
	$PM_{2.5}$ (μg/m³)	**Non-linear[1]; Fig. 1b**	—	**<0.001**	**<0.001**
	Total road density (per 1000 m/km²; 200-m buffer)	*b* = 0.054	−0.233, 0.340	0.713	0.713
	Major road density (per 1000 m/km²; 200-m buffer)	*b* = −0.250	−0.606, 0.107	0.170	0.283
	Distance to nearest busy road (per 100 m)	*b* = 0.059	−0.075, 0.193	0.391	0.489
Diastolic BP (mmHg)	NO_2 (ppb)	***b* = −0.348**	**−0.579, −0.117**	**0.003**	**0.009**
	$PM_{2.5}$ (μg/m³)	***b* = 0.428**	**0.195, 0.661**	**<0.001**	**<0.001**
	Total road density (per 1000 m/km²; 200-m buffer)	*b* = 0.002	−0.170, 0.175	0.978	0.978
	Major road density (per 1000 m/km²; 200-m buffer)	*b* = −0.026	−0.240, 0.189	0.813	0.978
	Distance to nearest busy road (per 100 m)	*b* = −0.025	−0.105, 0.054	0.533	0.888
HDL-C (mmol/L)	NO_2 (ppb)	*b* = −0.001	−0.010, 0.007	0.753	0.776
	$PM_{2.5}$ (μg/m³)	*b* = −0.007	−0.016, 0.002	0.117	0.293
	Total road density (per 1000 m/km²; 200-m buffer)	***b* = −0.007**	**−0.013, −0.001**	**0.030**	0.150
	Major road density (per 1000 m/km²; 200-m buffer)	*b* = −0.004	−0.012, 0.004	0.353	0.588
	Distance to nearest busy road (per 100 m)	*b* = 0.0004	−0.003, 0.003	0.776	0.776
LDL-C (mmol/L)	NO_2 (ppb)	*b* = −0.010	−0.028, 0.008	0.266	0.657
	$PM_{2.5}$ (μg/m³)	***b* = 0.018**	**0.0003, 0.035**	**0.047**	0.235
	Total road density (per 1000 m/km²; 200-m buffer)	*b* = −0.004	−0.018, 0.009	0.526	0.658
	Major road density (per 1000 m/km²; 200-m buffer)	*b* = −0.003	−0.020, 0.014	0.767	0.767
	Distance to nearest busy road (per 100 m)	*b* = −0.002	−0.008, 0.004	0.501	0.658
Triglycerides (mmol/L)	NO_2 (ppb)	e^b = 1.001	0.989, 1.013	0.847	0.847
	$PM_{2.5}$ (μg/m³)	e^b = 1.009	0.998, 1.021	0.123	0.175
	Total road density (per 1000 m/km²; 200-m buffer)	**e^b = 1.010**	**1.001, 1.020**	**0.031**	0.105

CMB health outcome	Environmental indicator	Estimate/statistic	95% CI	*p*	*q*
	Major road density (per 1000 m/km^2; 200-m buffer)	**e^b = 1.012**	**1.000, 1.024**	**0.042**	0.105
	Distance to nearest busy road (per 100 m)	e^b = 0.997	0.993, 1.001	0.140	0.175
HbA1c (mmol/mol)	NO_2 (ppb)	**e^b = 1.004**	**1.001, 1.008**	**0.020**	0.050
	$PM_{2.5}$ (μg/m^3)	e^b = 1.002	0.998, 1.005	0.337	0.562
	Total road density (per 1000 m/km^2; 200-m buffer)	e^b = 1.000	0.998, 1.002	0.998	0.998
	Major road density (per 1000 m/km^2; 200-m buffer)	e^b = 1.000	0.997, 1.003	0.831	0.998
	Distance to nearest busy road (per 100 m)	**Non-linear[1]; Fig. 1d**	**—**	**<0.001**	**0.002**

Notes: Road-density estimates refer to measures calculated within 200-m Euclidean residential buffers. $PM_{2.5}$ and NO_2 were estimated at participants' residential addresses, and aerial distance to the nearest busy road was calculated from the residential address; these three indicators were not defined using residential buffers. For these sensitivity analyses, buffer-based neighborhood covariates were specified using 200-m residential buffers. GAMMs with Gaussian variance and identity link functions were used for waist circumference, systolic and diastolic BP, HDL-C and LDL-C. GAMMs with Gamma variance and logarithmic link functions were used for triglycerides and HbA1c. All models were adjusted for the covariates specified in Table S.3.1. b, regression coefficient; e^b, exponentiated regression coefficient; CI, confidence interval; BP, blood pressure; HDL-C, high-density lipoprotein cholesterol; LDL-C, low-density lipoprotein cholesterol; HbA1c, glycated hemoglobin; NO_2, nitrogen dioxide; $PM_{2.5}$, particulate matter with an aerodynamic diameter ≤2.5 μm. *p*, *p*-value; *q*, Benjamini–Hochberg false discovery rate (FDR)-adjusted p-value. Bold *p*-values indicate $p < 0.05$; bold *q*-values indicate $q < 0.05$. [1] Pooled *p*-value for the smooth term obtained using a D1 multivariate Wald test based on Rubin's multiple-imputation combining rules.

The shapes of the non-linear associations of NO_2 and $PM_{2.5}$ with systolic BP, and of distance to the nearest busy road with HbA1c, were virtually identical to those presented in the main manuscript and are therefore not reproduced here.

Table S.6.2. Sensitivity analyses of associations of ambient air pollution and road-related environmental indicators with cardiometabolic health outcomes: 500-m residential buffer specification.

CMB health outcome	Environmental indicator	Estimate/statistic	95% CI	p	q
Waist circumference (cm)	NO_2 (ppb)	b = 0.066	−0.244, 0.375	0.678	0.678
	$PM_{2.5}$ (μg/m^3)	**b = 0.344**	**0.046, 0.643**	**0.024**	0.120
	Total road density (per 1000 m/km^2; 500-m buffer)	b = 0.267	−0.048, 0.581	0.097	0.230
	Major road density (per 1000 m/km^2; 500-m buffer)	b = 0.136	−0.234, 0.505	0.472	0.590
	Distance to nearest busy road (per 100 m)	b = −0.077	−0.178, 0.025	0.138	0.230
Systolic BP (mmHg)	NO_2 (ppb)	**Non-linear[1]; Fig. 1a**	—	**<0.001**	**<0.001**
	$PM_{2.5}$ (μg/m^3)	**Non-linear[1]; Fig. 1b**	—	**<0.001**	**<0.001**
	Total road density (per 1000 m/km^2; 500-m buffer)	b = −0.230	−0.646, 0.187	0.280	0.280
	Major road density (per 1000 m/km^2; 500-m buffer)	**Non-linear[1]; Fig. S.6.1**	—	**0.043**	0.072
	Distance to nearest busy road (per 100 m)	b = 0.092	−0.042, 0.226	0.177	0.221
Diastolic BP (mmHg)	NO_2 (ppb)	**b = −0.325**	**−0.567, −0.083**	**0.009**	**0.023**
	$PM_{2.5}$ (μg/m^3)	**b = 0.418**	**0.184, 0.653**	**<0.001**	**0.003**
	Total road density (per 1000 m/km^2; 500-m buffer)	b = −0.071	−0.321, 0.179	0.578	0.723
	Major road density (per 1000 m/km^2; 500-m buffer)	b = −0.113	−0.406, 0.179	0.447	0.723
	Distance to nearest busy road (per 100 m)	b = −0.004	−0.084, 0.076	0.927	0.927
HDL-C (mmol/L)	NO_2 (ppb)	b = −0.002	−0.011, 0.007	0.646	0.717
	$PM_{2.5}$ (μg/m^3)	b = −0.008	−0.017, 0.001	0.088	0.220
	Total road density (per 1000 m/km^2; 500-m buffer)	**b = −0.014**	**−0.024, −0.005**	**0.003**	**0.015**
	Major road density (per 1000 m/km^2; 500-m buffer)	b = −0.002	−0.013, 0.009	0.692	0.717
	Distance to nearest busy road (per 100 m)	b = 0.001	−0.002, 0.004	0.717	0.717
LDL-C (mmol/L)	NO_2 (ppb)	b = −0.007	−0.026, 0.012	0.455	0.569
	$PM_{2.5}$ (μg/m^3)	**b = 0.020**	**0.002, 0.038**	**0.028**	0.140
	Total road density (per 1000 m/km^2; 500-m buffer)	b = −0.006	−0.025, 0.014	0.577	0.577
	Major road density (per 1000 m/km^2; 500-m buffer)	b = 0.013	−0.010, 0.036	0.257	0.569
	Distance to nearest busy road (per 100 m)	b = −0.002	−0.009, 0.004	0.428	0.569
Triglycerides (mmol/L)	NO_2 (ppb)	e^b = 0.999	0.986, 1.012	0.856	0.856
	$PM_{2.5}$ (μg/m^3)	e^b = 1.007	0.995, 1.019	0.288	0.360
	Total road density (per 1000 m/km^2; 500-m buffer)	**e^b = 1.025**	**1.012, 1.038**	**<0.001**	**<0.001**
	Major road density (per 1000 m/km^2; 500-m buffer)	**e^b = 1.022**	**1.007, 1.038**	**0.005**	**0.013**

CMB health outcome	Environmental indicator	Estimate/statistic	95% CI	*p*	*q*
	Distance to nearest busy road (per 100 m)	$e^b = 0.998$	0.994, 1.002	0.260	0.360
HbA1c (mmol/mol)	NO_2 (ppb)	$e^b = 1.003$	0.999, 1.007	0.125	0.313
	$PM_{2.5}$ (µg/m^3)	$e^b = 1.001$	0.997, 1.005	0.540	0.652
	Total road density (per 1000 m/km^2; 500-m buffer)	$e^b = 1.001$	0.997, 1.005	0.652	0.652
	Major road density (per 1000 m/km^2; 500-m buffer)	$e^b = 1.002$	0.998, 1.007	0.330	0.550
	Distance to nearest busy road (per 100 m)	**Non-linear[1]; Fig. 1d**	—	**<0.001**	**<0.001**

Notes: Road-density estimates refer to measures calculated within 500-m Euclidean residential buffers. $PM_{2.5}$ and NO_2 were estimated at participants' residential addresses, and aerial distance to the nearest busy road was calculated from the residential address; these three indicators were not defined using residential buffers. For these sensitivity analyses, buffer-based neighborhood covariates were specified using 500-m residential buffers. GAMMs with Gaussian variance and identity link functions were used for waist circumference, systolic and diastolic BP, HDL-C and LDL-C. GAMMs with Gamma variance and logarithmic link functions were used for triglycerides and HbA1c. All models were adjusted for the covariates specified in Table S.3.1. *b*, regression coefficient; e^b, exponentiated regression coefficient; CI, confidence interval; BP, blood pressure; HDL-C, high-density lipoprotein cholesterol; LDL-C, low-density lipoprotein cholesterol; HbA1c, glycated hemoglobin; NO_2, nitrogen dioxide; $PM_{2.5}$, particulate matter with an aerodynamic diameter ≤2.5 µm. *p*, *p*-value; *q*, Benjamini–Hochberg false discovery rate (FDR)-adjusted *p*-value. Bold *p*-values indicate $p < 0.05$; bold *q*-values indicate $q < 0.05$. [1] Pooled *p*-value for the smooth term obtained using a D1 multivariate Wald test based on Rubin's multiple-imputation combining rules.

The shapes of the non-linear associations of NO_2 and $PM_{2.5}$ with systolic BP, and of distance to the nearest busy road with HbA1c, were virtually identical to those presented in the main manuscript and are therefore not reproduced here.

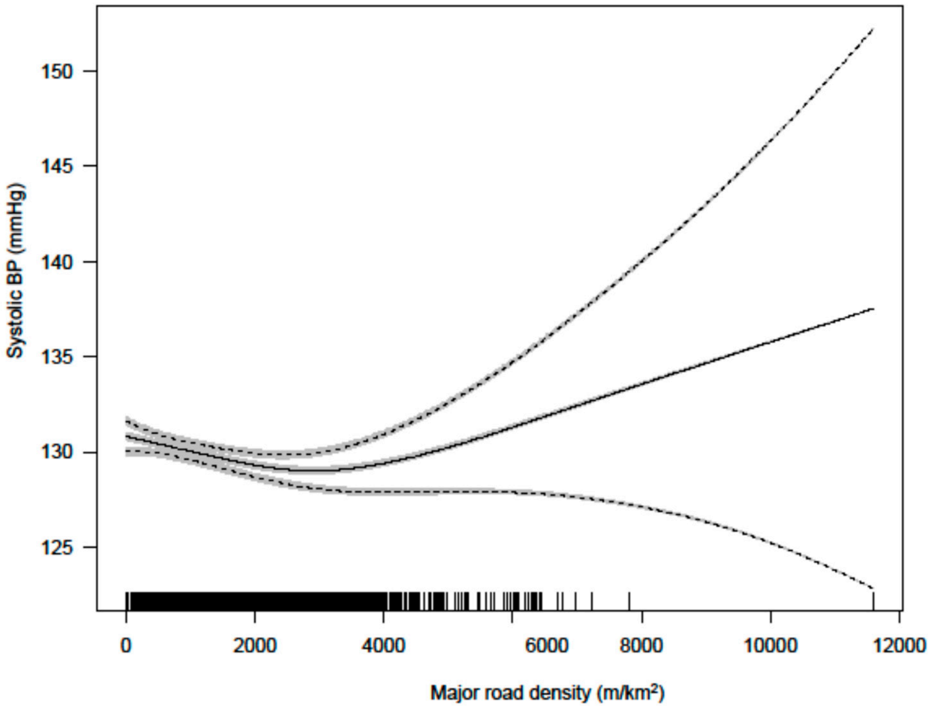

Figure S.6.1. Estimated non-linear association of major road density (500-m buffer) with systolic blood pressure (did not meet the FDR-adjusted significance level, $q < 0.05$).

S.7. Associations involving walking used to evaluate hypothesized behavioral relationships between environmental indicators and cardiometabolic health

Table S.7.1. Primary analyses of associations involving total walking used to evaluate hypothesized behavioral relationships between environmental indicators and cardiometabolic health (1-km spatial specification).

A. Environmental indicator–walking associations

Environmental indicator	Participation in total walking (yes/no)				Non-zero total walking time (min/week)			
	**OR**	**95% CI**	***p***	***q***	**e^b**	**95% CI**	***p***	***q***
NO_2 (ppb)	1.057	0.991, 1.127	0.091	0.152	0.996	0.973, 1.019	0.729	0.729
$PM_{2.5}$ (μg/m^3)	0.951	0.898, 1.007	0.083	0.152	0.981	0.960, 1.002	0.076	0.095
Total road density (per 1000 m/km^2; 1-km buffer)	0.910	0.841, 0.985	**0.019**	0.095	0.939	0.913, 0.966	**<0.001**	**<0.001**
Major road density (per 1000 m/km^2; 1-km buffer)	0.925	0.829, 1.033	0.165	0.206	0.953	0.912, 0.994	**0.027**	**0.045**
Distance to nearest busy road (per 100 m)	1.001	0.982, 1.020	0.947	0.947	1.010	1.003, 1.017	**0.006**	**0.015**

B. CMB health models including total walking

CMB health outcome	Environmental indicator	Total walking participation & CMB adj. for environmental indicator	Total walking time & CMB adj. for environmental indicator	Environmental indicator & CMB adj. for both total walking variables	JS: walking participation	JS: walking time
Waist circumference (cm)	NO_2 (ppb)	-1.790 (-2.885, -0.695)<br>***p* = 0.001; *q* = 0.001**	-0.006 (-0.008, -0.004)<br>***p* < 0.001; *q* < 0.001**	0.080 (-0.245, 0.404)<br>*p* = 0.630; *q* = 0.630	—	—
	$PM_{2.5}$ (μg/m^3)	-1.774 (-2.868, -0.680)<br>***p* = 0.001; *q* = 0.001**	-0.006 (-0.008, -0.004)<br>***p* < 0.001; *q* < 0.001**	0.316 (0.014, 0.618)<br>***p* = 0.040;** *q* = 0.201	—	—
	Total road density (per 1000 m/km^2; 1-km buffer)	-1.786 (-2.881, -0.692)<br>***p* = 0.001; *q* = 0.001**	-0.006 (-0.008, -0.004)<br>***p* < 0.001; *q* < 0.001**	0.264 (-0.127, 0.655)<br>*p* = 0.185; *q* = 0.327	—	Met
	Major road density (per 1000 m/km^2; 1-km buffer)	-1.783 (-2.877, -0.688)<br>***p* = 0.001; *q* = 0.001**	-0.006 (-0.008, -0.004)<br>***p* < 0.001; *q* < 0.001**	0.260 (-0.319, 0.838)<br>*p* = 0.379; *q* = 0.474	—	Met
	Distance to nearest busy road (per 100 m)	-1.797 (-2.892, -0.703)<br>***p* = 0.001; *q* = 0.001**	-0.006 (-0.008, -0.004)<br>***p* < 0.001; *q* < 0.001**	-0.066 (-0.167, 0.034)<br>*p* = 0.196; *q* = 0.327	—	Met

CMB health outcome	Environmental indicator	Total walking participation & CMB adj. for environmental indicator	Total walking time & CMB adj. for environmental indicator	Environmental indicator & CMB adj. for both total walking variables	JS: walking participation	JS: walking time
Systolic BP (mmHg)	NO_2 (ppb)	-0.048 (-1.516, 1.420) *p* = 0.949; *q* = 0.949	0.0004 (-0.003, 0.003) *p* = 0.783; *q* = 0.836	**Non-linear[1] *p* = 0.002; *q* = 0.005 (Fig. S.7.1a)**	—	—
	$PM_{2.5}$ (μg/m³)	-0.089 (-1.557, 1.379) *p* = 0.906; *q* = 0.949	0.0005 (-0.002, 0.003) *p* = 0.758; *q* = 0.836	**Non-linear[1] *p* < 0.001; *q* < 0.001 (Fig. S.7.1b)**	—	—
	Total road density (per 1000 m/km²; 1-km buffer)	-0.090 (-1.558, 1.378) *p* = 0.904; *q* = 0.949	0.0003 (-0.003, 0.003) *p* = 0.836; *q* = 0.836	-0.332 (-0.847, 0.183) *p* = 0.206; *q* = 0.258	—	—
	Major road density (per 1000 m/km²; 1-km buffer)	-0.094 (-1.563, 1.374) *p* = 0.900; *q* = 0.949	0.0004 (-0.003, 0.003) *p* = 0.784; *q* = 0.836	-0.193 (-0.956, 0.570) *p* = 0.620; *q* = 0.620	—	—
	Distance to nearest busy road (per 100 m)	-0.072 (-1.540, 1.397) *p* = 0.924; *q* = 0.949	0.0003 (-0.003, 0.003) *p* = 0.817; *q* = 0.836	0.098 (-0.033, 0.230) *p* = 0.141; *q* = 0.235	—	—
Diastolic BP (mmHg)	NO_2 (ppb)	-0.298 (-1.196, 0.600) *p* = 0.515; *q* = 0.515	-0.001 (-0.003, 0.001) *p* = 0.262; *q* = 0.303	-0.258 (-0.510, -0.006) ***p* = 0.045;** *q* = 0.113	—	—
	$PM_{2.5}$ (μg/m³)	-0.308 (-1.204, 0.589) *p* = 0.501; *q* = 0.515	-0.001 (-0.003, 0.001) *p* = 0.303; *q* = 0.303	0.366 (0.131, 0.602) ***p* = 0.002; *q* = 0.011**	—	—
	Total road density (per 1000 m/km²; 1-km buffer)	-0.321 (-1.219, 0.577) *p* = 0.483; *q* = 0.515	-0.001 (-0.003, 0.001) *p* = 0.259; *q* = 0.303	-0.045 (-0.353, 0.263) *p* = 0.775; *q* = 0.825	—	—
	Major road density (per 1000 m/km²; 1-km buffer)	-0.320 (-1.218, 0.577) *p* = 0.484; *q* = 0.515	-0.001 (-0.003, 0.001) *p* = 0.270; *q* = 0.303	0.051 (-0.403, 0.505) *p* = 0.825; *q* = 0.825	—	—
	Distance to nearest busy road (per 100 m)	-0.319 (-1.217, 0.579) *p* = 0.486; *q* = 0.515	-0.001 (-0.003, 0.001) *p* = 0.262; *q* = 0.303	0.011 (-0.067, 0.089) *p* = 0.786; *q* = 0.825	—	—
HDL-C (mmol/L)	NO_2 (ppb)	0.015 (-0.019, 0.048) *p* = 0.390; *q* = 0.408	0.0002 (0.0001, 0.0003) ***p* < 0.001; *q* < 0.001**	-0.005 (-0.014, 0.005) *p* = 0.343; *q* = 0.565	—	—
	$PM_{2.5}$ (μg/m³)	0.014 (-0.019, 0.047) *p* = 0.408; *q* = 0.408	0.0002 (0.0001, 0.0003) ***p* < 0.001; *q* < 0.001**	-0.006 (-0.015, 0.003) *p* = 0.175; *q* = 0.438	—	—
	Total road density (per 1000 m/km²; 1-km buffer)	0.014 (-0.019, 0.047) *p* = 0.401; *q* = 0.408	0.0002 (0.0001, 0.0003) ***p* < 0.001; *q* < 0.001**	-0.016 (-0.028, -0.004) ***p* = 0.007; *q* = 0.035**	—	Met

CMB health outcome	Environmental indicator	Total walking participation & CMB adj. for environmental indicator	Total walking time & CMB adj. for environmental indicator	Environmental indicator & CMB adj. for both total walking variables	JS: walking participation	JS: walking time
	Major road density (per 1000 m/km²; 1-km buffer)	0.014 (-0.019, 0.047) *p* = 0.404; *q* = 0.408	0.0002 (0.0001, 0.0003) ***p* < 0.001; *q* < 0.001**	-0.007 (-0.024 0.011) *p* = 0.452; *q* = 0.565	—	Met
	Distance to nearest busy road (per 100 m)	0.014 (-0.019, 0.047) *p* = 0.400; *q* = 0.408	0.0002 (0.0001,0.0003) ***p* < 0.001; *q* < 0.001**	0.0003 (-0.003, 0.003) *p* = 0.854; *q* = 0.854	—	Met
LDL-C (mmol/L)	NO_2 (ppb)	-0.044 (-0.119, 0.030) *p* = 0.242; *q* = 0.249	0.0002 (0.000, 0.0004) ***p* = 0.002; *q* = 0.003**	-0.003 (-0.023, 0.017) *p* = 0.756; *q* = 0.945	—	—
	$PM_{2.5}$ (μg/m³)	-0.044 (-0.118, 0.031) *p* = 0.249; *q* = 0.249	0.0002 (0.000, 0.0004) ***p* = 0.002; *q* = 0.003**	0.021 (0.002, 0.039) ***p* = 0.027;** *q* = 0.135	—	—
	Total road density (per 1000 m/km²; 1-km buffer)	-0.045 (-0.119, 0.030) *p* = 0.238; *q* = 0.249	0.0002 (0.000, 0.0004) ***p* = 0.003; *q* = 0.003**	-0.0004 (-0.025, 0.024) *p* = 0.976; *q* = 0.976	—	Met
	Major road density (per 1000 m/km²; 1-km buffer)	-0.045 (-0.119, 0.030) *p* = 0.240; *q* = 0.249	0.0002 (0.000, 0.0004) ***p* = 0.002; *q* = 0.003**	0.019 (-0.016, 0.055) *p* = 0.291; *q* = 0.728	—	Met
	Distance to nearest busy road (per 100 m)	-0.045 (-0.119, 0.029) *p* = 0.235; *q* = 0.249	0.0002 (0.0001, 0.0004) ***p* = 0.002; *q* = 0.003**	-0.002 (-0.008, 0.004) *p* = 0.469; *q* = 0.782	—	Met
Triglycerides (mmol/L)	NO_2 (ppb)	0.981 (0.931, 1.033) *p* = 0.458; *q* = 0.495	0.9999 (0.9998, 0.9999) ***p* = 0.012; *q* = 0.021**	1.000 (0.987, 1.013) *p* = 0.982; *q* = 0.982	—	—
	$PM_{2.5}$ (μg/m³)	0.981 (0.931, 1.033) *p* = 0.463; *q* = 0.495	0.9998 (0.9997, 0.9999) ***p* = 0.014; *q* = 0.021**	1.004 (0.992, 1.017) *p* = 0.505; *q* = 0.631	—	—
	Total road density (per 1000 m/km²; 1-km buffer)	0.981 (0.931, 1.034) *p* = 0.473; *q* = 0.495	0.9998 (0.9997, 0.9999) ***p* = 0.026; *q* = 0.026**	1.030 (1.013, 1.047) ***p* < 0.001; *q* = 0.001**	—	Met
	Major road density (per 1000 m/km²; 1-km buffer)	0.982 (0.932, 1.034) *p* = 0.495; *q* = 0.495	0.9998 (0.9997, 0.9999) ***p* = 0.016; *q* = 0.021**	1.040 (1.015, 1.065) ***p* = 0.001; *q* = 0.003**	—	Met
	Distance to nearest busy road (per 100 m)	0.980 (0.931, 1.033) *p* = 0.454; *q* = 0.495	0.9998 (0.9997, 0.9999) ***p* = 0.015; *q* = 0.021**	0.998 (0.994, 1.002) *p* = 0.354; *q* = 0.591	—	Met
HbA1c (mmol/mol)	NO_2 (ppb)	0.986 (0.974, 0.998) ***p* = 0.024; *q* = 0.030**	0.9999 (0.9999, 1.000) *p* = 0.094; *q* = 0.103	1.002 (0.998, 1.006) *p* = 0.310; *q* = 0.517	—	—
	$PM_{2.5}$ (μg/m³)	0.986 (0.974, 0.998) ***p* = 0.025; *q* = 0.030**	0.9999 (0.9999, 1.000) *p* = 0.095; *q* = 0.103	1.001 (0.997, 1.004) *p* = 0.704; *q* = 0.704	—	—

CMB health outcome	Environmental indicator	Total walking participation & CMB adj. for environmental indicator	Total walking time & CMB adj. for environmental indicator	Environmental indicator & CMB adj. for both total walking variables	JS: walking participation	JS: walking time
	Total road density (per 1000 m/km²; 1-km buffer)	0.986 (0.974, 0.998) **p = 0.025; q = 0.030**	0.9999 (0.9999, 1.000) p = 0.100; q = 0.103	1.001 (0.997, 1.006) p = 0.610; q = 0.704	—	—
	Major road density (per 1000 m/km²; 1-km buffer)	0.986 (0.974, 0.998) **p = 0.025; q = 0.030**	0.9999 (0.9999, 1.000) p = 0.103; q = 0.103	**Non-linear[1] p = 0.002; q = 0.005 (Fig. S.7.1c)**	—	—
	Distance to nearest busy road (per 100 m)	0.986 (0.974, 0.999) p = **0.030; q = 0.030**	0.9999 (0.9999, 1.000) p = 0.073; q = 0.103	**Non-linear[1] p < 0.001; q < 0.001 (Fig. S.7.1d)**	—	—

Notes: The joint-significance criterion was considered met when both the environmental indicator–walking association and the corresponding walking–CMB association adjusted for the environmental indicator had FDR-adjusted q-values < 0.05. A met JS criterion indicates a cross-sectional pattern of component associations consistent with a hypothesized behavioral pathway; it does not establish temporal ordering, an indirect effect or causal mediation. The environmental indicator–CMB association adjusted for walking is reported descriptively and should not be interpreted as a direct causal effect; it was not used to determine the JS criterion. Participation in total walking was modelled using a binomial GAMM with logit link; non-zero total walking time was modelled using a Gamma GAMM with logarithmic link. For waist circumference, systolic and diastolic BP, HDL-C and LDL-C, CMB-model estimates are regression coefficients (b). For triglycerides and HbA1c, estimates are exponentiated regression coefficients (e^b). CI, confidence interval; CMB, cardiometabolic; BP, blood pressure; HDL-C, high-density lipoprotein cholesterol; LDL-C, low-density lipoprotein cholesterol; HbA1c, glycated hemoglobin; adj., adjusted. p, p-value; q, Benjamini–Hochberg false discovery rate (FDR)-adjusted p-value. Bold p-values indicate p < 0.05; bold q-values indicate q < 0.05. [1] Pooled p-value for the smooth term obtained using a D1 multivariate Wald test based on Rubin's multiple-imputation combining rules.

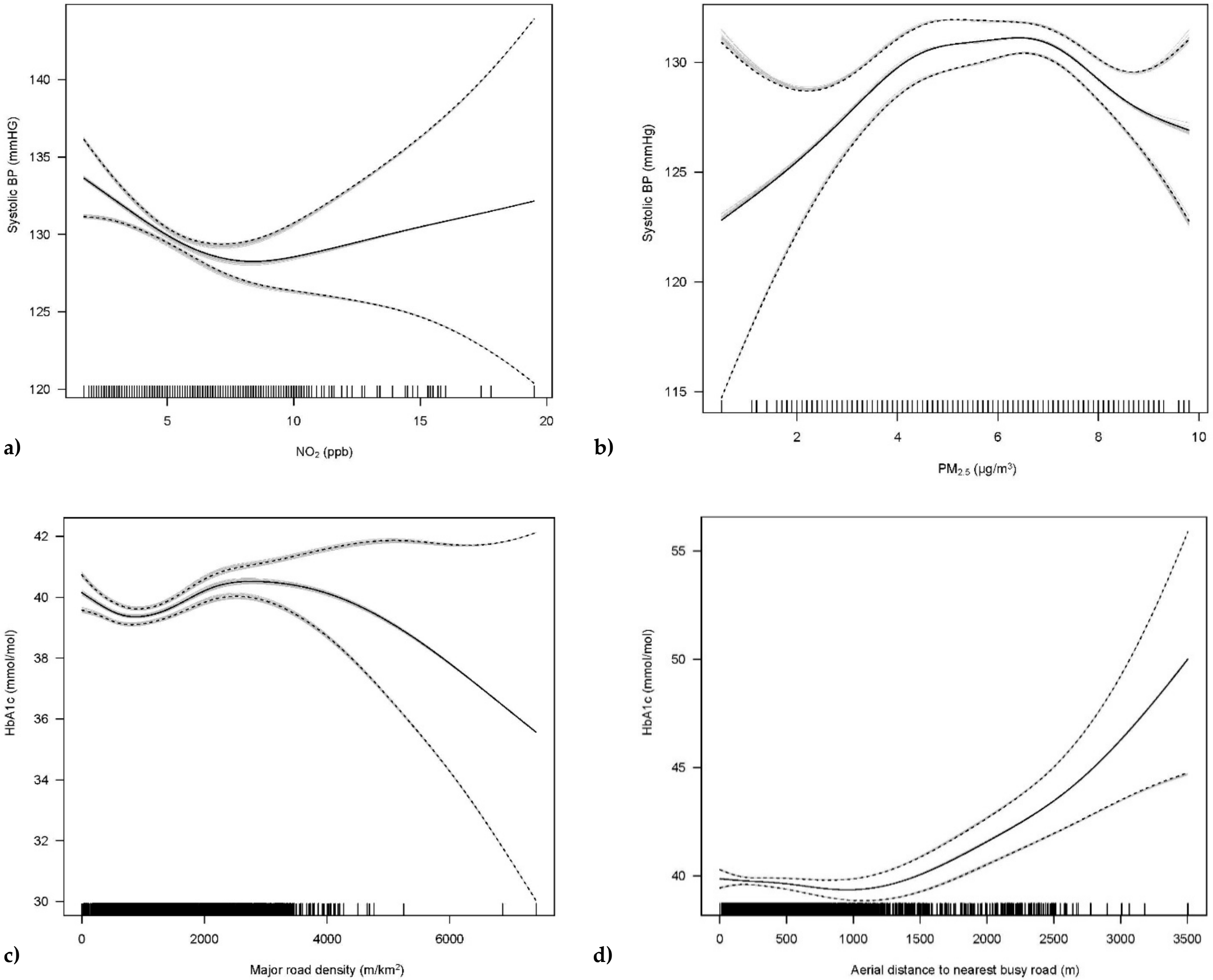

Figure S.7.1. Total walking-adjusted non-linear associations of (a) NO_2 with systolic blood pressure, (b) $PM_{2.5}$ with systolic blood pressure, (c) major road density with HbA1c, and (d) aerial distance to the nearest busy road with HbA1c.

Table S.7.2. Primary analyses of associations involving walking for transport used to evaluate hypothesized behavioral relationships between environmental indicators and cardiometabolic health (1-km spatial specification).

A. Environmental indicator–walking associations

**Environmental indicator**	**Participation in walking for transport (yes/no)**				**Non-zero walking-for-transport frequency (times/week)**			
	**OR**	**95% CI**	***p***	***q***	**e^b**	**95% CI**	***p***	***q***
NO_2 (ppb)	1.106	1.046, 1.170	**<0.001**	**<0.001**	1.010	0.986, 1.034	0.412	0.437
$PM_{2.5}$ (μg/m³)	1.116	1.057, 1.179	**<0.001**	**<0.001**	1.011	0.986, 1.038	0.386	0.437
Total road density (per 1000 m/km²; 1-km buffer)	1.039	0.966, 1.116	0.302	0.302	1.024	0.993, 1.055	0.128	0.437
Major road density (per 1000 m/km²; 1-km buffer)	1.077	0.972, 1.194	0.158	0.198	1.023	0.976, 1.073	0.340	0.437
Distance to nearest busy road (per 100 m)	0.979	0.961, 0.998	**0.029**	**0.048**	1.003	0.995, 1.012	0.437	0.437

B. CMB health models including walking for transport

**CMB health outcome**	**Environmental indicator**	**Walking for transport participation & CMB adj. for environmental indicator**	**Walking-for-transport frequency & CMB adj. for environmental indicator**	**Environmental indicator & CMB adj. for both walking for transport variables**	**JS: walking participation**	**JS: walking frequency**
Waist circumference (cm)	NO_2 (ppb)	-2.147 (-3.656, -0.638)<br>***p* = 0.005; *q* = 0.006**	0.046 (-0.281, 0.372)<br>*p* = 0.784; *q* = 0.792	0.098 (-0.230, 0.427)<br>*p* = 0.559; *q* = 0.559	Met	—
	$PM_{2.5}$ (μg/m³)	-2.195 (-3.703, -0.687)<br>***p* = 0.004; *q* = 0.006**	0.045 (-0.281, 0.371)<br>*p* = 0.787; *q* = 0.792	0.399 (0.093, 0.705)<br>***p* = 0.011**; *q* = 0.055	Met	—
	Total road density (per 1000 m/km²; 1-km buffer)	-2.141 (-3.649, -0.633)<br>***p* = 0.005; *q* = 0.006**	0.044 (-0.282, 0.370)<br>*p* = 0.789; *q* = 0.792	0.375 (-0.019, 0.769)<br>*p* = 0.062; *q* = 0.155	—	—
	Major road density (per 1000 m/km²; 1-km buffer)	-2.135 (-3.644, -0.627)<br>***p* = 0.006; *q* = 0.006**	0.044 (-0.282, 0.370)<br>*p* = 0.792; *q* = 0.792	0.361 (-0.224, 0.945)<br>*p* = 0.227; *q* = 0.284	—	—
	Distance to nearest busy road (per 100 m)	-2.172 (-3.682, -0.663)<br>***p* = 0.005; *q* = 0.006**	0.050 (-0.276, 0.376)<br>*p* = 0.762; *q* = 0.792	-0.083 (-0.185, 0.019)<br>*p* = 0.109; *q* = 0.182	Met	—

CMB health outcome	Environmental indicator	Walking for transport participation & CMB adj. for environmental indicator	Walking-for-transport frequency & CMB adj. for environmental indicator	Environmental indicator & CMB adj. for both walking for transport variables	JS: walking participation	JS: walking frequency
Systolic BP (mmHg)	NO_2 (ppb)	-0.533 (-2.556, 1.489) *p* = 0.605; *q* = 0.605	0.016 (-0.421, 0.452) *p* = 0.944; *q* = 0.985	**Non-linear[1]** ***p* = 0.002; *q* = 0.005** **(Fig. S.7.2a)**	—	—
	$PM_{2.5}$ (μg/m³)	-0.605 (-2.630, 1.419) *p* = 0.558; *q* = 0.605	0.009 (-0.428, 0.445) *p* = 0.969; *q* = 0.985	**Non-linear[1]** ***p* < 0.001; *q* < 0.001** **(Fig. S.7.2b)**	—	—
	Total road density (per 1000 m/km²; 1-km buffer)	-0.580 (-2.604, 1.443) *p* = 0.574; *q* = 0.605	0.011 (-0.425, 0.447) *p* = 0.961; *q* = 0.985	-0.332 (-0.846, 0.181) *p* = 0.204; *q* = 0.255	—	—
	Major road density (per 1000 m/km²; 1-km buffer)	-0.583 (-2.607, 1.440) *p* = 0.572; *q* = 0.605	0.011 (-0.426, 0.447) *p* = 0.962; *q* = 0.985	-0.190 (-0.952, 0.572) *p* = 0.625; *q* = 0.625	—	—
	Distance to nearest busy road (per 100 m)	-0.539 (-2.563, 1.485) *p* = 0.602; *q* = 0.605	0.004 (-0.432, 0.441) *p* = 0.985; *q* = 0.985	0.098 (-0.034, 0.229) *p* = 0.146; *q* = 0.243	—	—
Diastolic BP (mmHg)	NO_2 (ppb)	-0.313 (-1.550, 0.923) *p* = 0.619; *q* = 0.619	0.061 (-0.205, 0.327) *p* = 0.651; *q* = 0.678	-0.261 (-0.513, -0.008) ***p* = 0.043;** *q* = 0.108	—	—
	$PM_{2.5}$ (μg/m³)	-0.407 (-1.644, 0.829) *p* = 0.518; *q* = 0.619	0.056 (-0.209, 0.322) *p* = 0.678; *q* = 0.678	0.377 (0.141, 0.613) ***p* = 0.002; *q* = 0.010**	—	—
	Total road density (per 1000 m/km²; 1-km buffer)	-0.341 (-1.578, 0.896) *p* = 0.589; *q* = 0.619	0.058 (-0.208, 0.324) *p* = 0.671; *q* = 0.678	-0.027 (-0.334, 0.280) *p* = 0.863; *q* = 0.863	—	—
	Major road density (per 1000 m/km²; 1-km buffer)	-0.341 (-1.578, 0.896) *p* = 0.589; *q* = 0.619	0.057 (-0.209, 0.323) *p* = 0.676; *q* = 0.678	0.066 (-0.388, 0.519) *p* = 0.776; *q* = 0.863	—	—
	Distance to nearest busy road (per 100 m)	-0.337 (-1.575, 0.901) *p* = 0.593; *q* = 0.619	0.057 (-0.209, 0.323) *p* = 0.675; *q* = 0.678	0.008 (-0.070, 0.086) *p* = 0.835; *q* = 0.863	—	—
HDL-C (mmol/L)	NO_2 (ppb)	0.059 (0.013, 0.105) ***p* = 0.013; *q* = 0.013**	-0.004 (-0.014, 0.006) *p* = 0.462; *q* = 0.470	-0.005 (-0.015, 0.004) *p* = 0.287; *q* = 0.359	Met	—
	$PM_{2.5}$ (μg/m³)	0.059 (0.013, 0.105) ***p* = 0.011; *q* = 0.013**	-0.004 (-0.014, 0.006) *p* = 0.456; *q* = 0.470	-0.008 (-0.017, 0.001) *p* = 0.076; *q* = 0.190	Met	—
	Total road density (per 1000 m/km²; 1-km buffer)	0.058 (0.012, 0.104) ***p* = 0.013; *q* = 0.013**	-0.004 (-0.014, 0.006) *p* = 0.470; *q* = 0.470	-0.019 (-0.031, -0.008) ***p* = 0.001; *q* = 0.005**	—	—

CMB health outcome	Environmental indicator	Walking for transport participation & CMB adj. for environmental indicator	Walking-for-transport frequency & CMB adj. for environmental indicator	Environmental indicator & CMB adj. for both walking for transport variables	JS: walking participation	JS: walking frequency
	Major road density (per 1000 m/km²; 1-km buffer)	0.058 (0.012, 0.104) **$p = 0.013$; $q = 0.013$**	-0.004 (-0.014, 0.006) $p = 0.462$; $q = 0.470$	-0.009 (-0.027 0.008) $p = 0.287$; $q = 0.359$	—	—
	Distance to nearest busy road (per 100 m)	0.058 (0.012, 0.104) **$p = 0.013$; $q = 0.013$**	-0.004 (-0.014, 0.006) $p = 0.448$; $q = 0.470$	0.001 (-0.002, 0.004) $p = 0.612$; $q = 0.612$	Met	—
LDL-C (mmol/L)	NO_2 (ppb)	-0.063 (-0.166, 0.040) $p = 0.233$; $q = 0.233$	0.025 (0.003, 0.047) **$p = 0.029$; $q = 0.031$**	-0.004 (-0.024, 0.016) $p = 0.681$; $q = 0.783$	—	—
	$PM_{2.5}$ (μg/m³)	-0.067 (-0.171, 0.036) $p = 0.202$; $q = 0.233$	0.025 (0.002, 0.047) **$p = 0.030$; $q = 0.031$**	0.019 (0.001, 0.038) **$p = 0.040$;** $q = 0.200$	—	—
	Total road density (per 1000 m/km²; 1-km buffer)	-0.063 (-0.167, 0.040) $p = 0.231$; $q = 0.233$	0.025 (0.002, 0.047) **$p = 0.030$; $q = 0.031$**	-0.003 (-0.028, 0.021) $p = 0.783$; $q = 0.783$	—	—
	Major road density (per 1000 m/km²; 1-km buffer)	-0.064 (-0.167, 0.040) $p = 0.228$; $q = 0.233$	0.025 (0.002, 0.047) **$p = 0.031$; $q = 0.031$**	0.016 (-0.020, 0.052) $p = 0.375$; $q = 0.783$	—	—
	Distance to nearest busy road (per 100 m)	-0.064 (-0.168, 0.039) $p = 0.225$; $q = 0.233$	0.025 (0.002, 0.047) **$p = 0.030$; $q = 0.031$**	-0.002 (-0.008, 0.004) $p = 0.554$; $q = 0.783$	—	—
Triglycerides (mmol/L)	NO_2 (ppb)	0.948 (0.883, 1.017) $p = 0.137$; $q = 0.137$	0.999 (0.984, 1.014) $p = 0.886$; $q = 0.909$	1.001 (0.988, 1.014) $p = 0.881$; $q = 0.881$	—	—
	$PM_{2.5}$ (μg/m³)	0.946 (0.882, 1.015) $p = 0.124$; $q = 0.137$	0.999 (0.984, 1.014) $p = 0.898$; $q = 0.909$	1.007 (0.995, 1.019) $p = 0.273$; $q = 0.341$	—	—
	Total road density (per 1000 m/km²; 1-km buffer)	0.947 (0.883, 1.016) $p = 0.131$; $q = 0.137$	0.999 (0.984, 1.014) $p = 0.896$; $q = 0.909$	1.032 (1.016, 1.049) **$p < 0.001$; $q < 0.001$**	—	—
	Major road density (per 1000 m/km²; 1-km buffer)	0.947 (0.883, 1.016) $p = 0.133$; $q = 0.137$	0.999 (0.984, 1.014) $p = 0.863$; $q = 0.909$	1.042 (1.018, 1.067) **$p < 0.001$; $q < 0.001$**	—	—
	Distance to nearest busy road (per 100 m)	0.946 (0.882, 1.015) $p = 0.125$; $q = 0.137$	0.999 (0.984, 1.015) $p = 0.909$; $q = 0.909$	0.998 (0.994, 1.002) $p = 0.221$; $q = 0.341$	—	—
HbA1c (mmol/mol)	NO_2 (ppb)	1.001 (0.984, 1.019); $p = 0.871$; $q = 0.871$	0.998 (0.995, 1.002) $p = 0.412$; $q = 0.421$	1.002 (0.998, 1.006) $p = 0.318$; $q = 0.530$	—	—
	$PM_{2.5}$ (μg/m³)	1.001 (0.984, 1.019) $p = 0.867$; $q = 0.871$	0.998 (0.995, 1.002) $p = 0.421$; $q = 0.421$	1.001 (0.997, 1.005) $p = 0.605$; $q = 0.605$	—	—

CMB health outcome	Environmental indicator	Walking for transport participation & CMB adj. for environmental indicator	Walking-for-transport frequency & CMB adj. for environmental indicator	Environmental indicator & CMB adj. for both walking for transport variables	JS: walking participation	JS: walking frequency
	Total road density (per 1000 m/km²; 1-km buffer)	1.002 (0.984, 1.019) $p = 0.859$; $q = 0.871$	0.998 (0.995, 1.002) $p = 0.419$; $q = 0.421$	1.002 (0.997, 1.006) $p = 0.486$; $q = 0.605$	—	—
	Major road density (per 1000 m/km²; 1-km buffer)	1.002 (0.984, 1.019) $p = 0.855$; $q = 0.871$	0.998 (0.995, 1.002) $p = 0.406$; $q = 0.421$	**Non-linear[1]** **$p = 0.001$; $q = 0.003$** **(Fig. S.7.2c)**	—	—
	Distance to nearest busy road (per 100 m)	1.002 (0.985, 1.020) $p = 0.801$; $q = 0.871$	0.998 (0.995, 1.002) $p = 0.401$; $q = 0.421$	**Non-linear[1]** **$p < 0.001$; $q < 0.001$** **(Fig. S.7.2d)**	—	—

Notes: The joint-significance criterion was considered met when both the environmental indicator–walking association and the corresponding walking–CMB association adjusted for the environmental indicator had FDR-adjusted *q*-values < 0.05. A met JS criterion indicates a cross-sectional pattern of component associations consistent with a hypothesized behavioral pathway; it does not establish temporal ordering, an indirect effect or causal mediation. The environmental indicator–CMB association adjusted for walking is reported descriptively and should not be interpreted as a direct causal effect; it was not used to determine the JS criterion. Participation in walking for transport was modelled using a binomial GAMM with logit link; non-zero walking-for-transport frequency was modelled using a negative-binomial GAMM with logarithmic link. For waist circumference, systolic and diastolic BP, HDL-C and LDL-C, CMB-model estimates are regression coefficients (*b*). For triglycerides and HbA1c, estimates are exponentiated regression coefficients (e^b). CI, confidence interval; CMB, cardiometabolic; BP, blood pressure; HDL-C, high-density lipoprotein cholesterol; LDL-C, low-density lipoprotein cholesterol; HbA1c, glycated hemoglobin; adj., adjusted. *p*, *p*-value; q, Benjamini–Hochberg false discovery rate (FDR)-adjusted *p*-value. Bold *p*-values indicate $p < 0.05$; bold *q*-values indicate $q < 0.05$. [1] Pooled *p*-value for the smooth term obtained using a D1 multivariate Wald test based on Rubin's multiple-imputation combining rules.

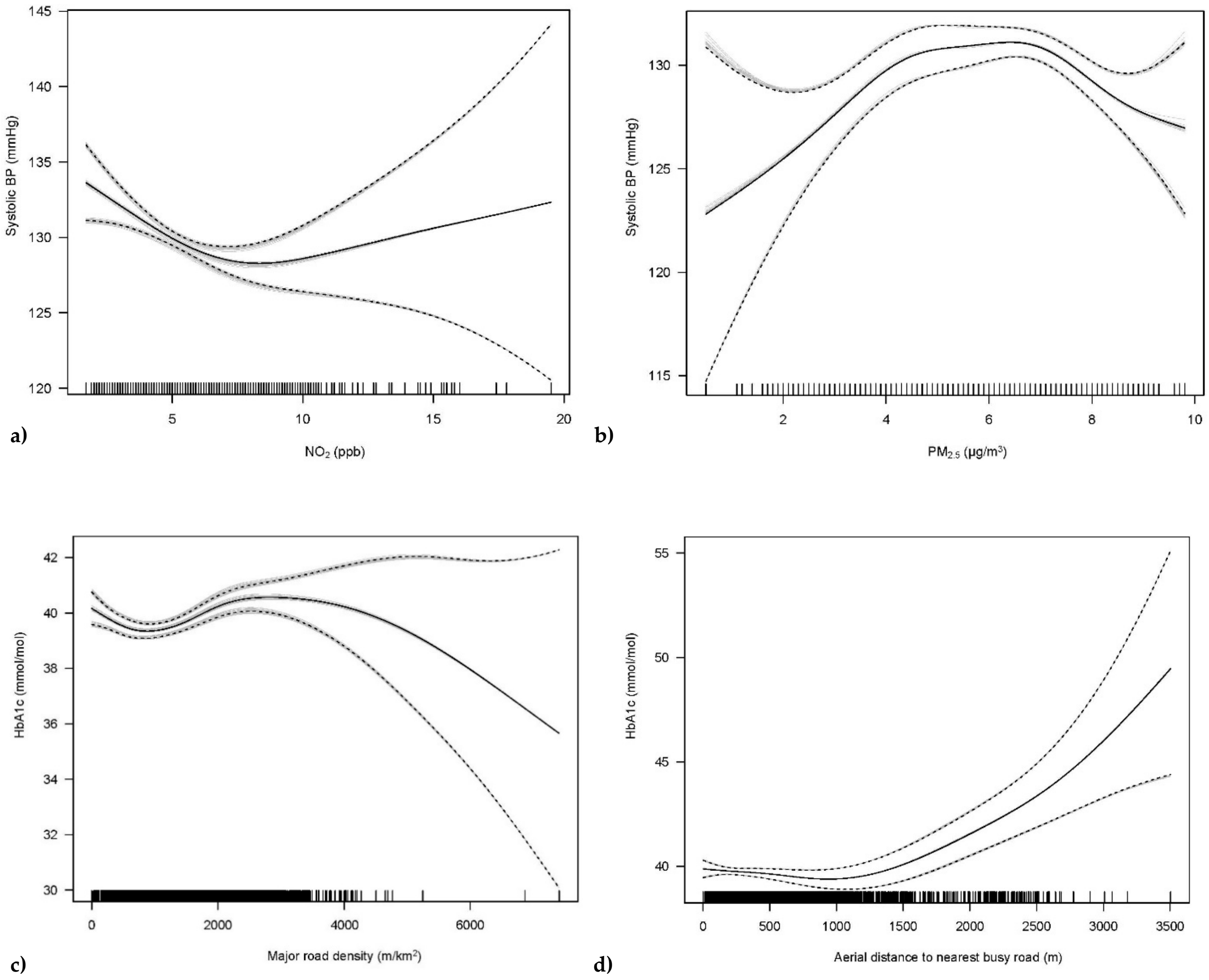

Figure S.7.2. Walking for transport-adjusted non-linear associations of (a) NO_2 with systolic blood pressure, (b) $PM_{2.5}$ with systolic blood pressure, (c) major road density with HbA1c, and (d) aerial distance to the nearest busy road with HbA1c.

Table S.7.3. Spatial-scale sensitivity analyses of associations involving total walking used to evaluate hypothesized behavioral relationships between environmental indicators and cardiometabolic health.

A. Environmental indicator–walking associations

Environmental indicator	Participation in total walking (yes/no)				Non-zero total walking time (min/week)			
	OR	95% CI	*p*	*q*	e^b	95% CI	*p*	*q*
NO_2 (ppb)	1.039	0.979, 1.103	0.205	0.256	0.990	0.969, 1.012	0.382	0.382
$PM_{2.5}$ (μg/m^3)	0.947	0.896, 1.000	0.052	0.203	0.978	0.958, 0.999	**0.041**	0.068
Total road density (per 1000 m/km^2; 500-m buffer)	0.949	0.893, 1.009	0.092	0.203	0.955	0.933, 0.977	**<0.001**	**<0.001**
Major road density (per 1000 m/km^2; 500-m buffer)	0.947	0.883, 1.015	0.122	0.203	0.974	0.948, 1.001	0.060	0.075
Distance to nearest busy road (per 100 m)	1.004	0.985, 1.023	0.664	0.664	1.010	1.003, 1.017	**0.005**	**0.013**

B. CMB health models including total walking

CMB health outcome	Environmental indicator	Total walking participation & CMB adj. for environmental indicator	Total walking time & CMB adj. for environmental indicator	Environmental indicator & CMB adj. for both total walking variables	JS: walking participation	JS: walking time
Waist circumference (cm)	NO_2 (ppb)	-1.766 (-2.861, -0.672)<br>**$p = 0.002$; $q = 0.002$**	-0.006 (-0.008, -0.004)<br>**$p < 0.001$; $q < 0.001$**	0.077 (-0.230, 0.383)<br>$p = 0.624$; $q = 0.627$	—	—
	$PM_{2.5}$ (μg/m^3)	-1.750 (-2.844, -0.656)<br>**$p = 0.002$; $q = 0.002$**	-0.006 (-0.008, -0.004)<br>**$p < 0.001$; $q < 0.001$**	0.299 (0.004, 0.595)<br>**$p = 0.047$;** $q = 0.235$	—	—
	Total road density (per 1000 m/km^2; 500-m buffer)	-1.767 (-2.861, -0.673)<br>**$p = 0.002$; $q = 0.002$**	-0.006 (-0.008, -0.004)<br>**$p < 0.001$; $q < 0.001$**	0.206 (-0.106, 0.518)<br>$p = 0.196$; $q = 0.330$	—	Met
	Major road density (per 1000 m/km^2; 500-m buffer)	-1.758 (-2.852, -0.664)<br>**$p = 0.002$; $q = 0.002$**	-0.006 (-0.008, -0.004)<br>**$p < 0.001$; $q < 0.001$**	0.091 (-0.275, 0.457)<br>$p = 0.627$; $q = 0.627$	—	—
	Distance to nearest busy road (per 100 m)	-1.771 (-2.866, -0.677)<br>**$p = 0.002$; $q = 0.002$**	-0.006 (-0.008, -0.004)<br>**$p < 0.001$; $q < 0.001$**	-0.066 (-0.166, 0.035)<br>$p = 0.198$; $q = 0.330$	—	Met
Systolic BP (mmHg)	NO_2 (ppb)	-0.014 (-1.485, 1.457)<br>$p = 0.985$; $q = 0.985$	0.0003 (-0.003, 0.003)<br>$p = 0.855$; $q = 0.876$	**Non-linear[1#]**<br>**$p < 0.001$, $q < 0.001$**<br>**(Fig. S.7.1a)**	—	—

CMB health outcome	Environmental indicator	Total walking participation & CMB adj. for environmental indicator	Total walking time & CMB adj. for environmental indicator	Environmental indicator & CMB adj. for both total walking variables	JS: walking participation	JS: walking time
	$PM_{2.5}$ (μg/m³)	-0.051 (-1.522, 1.421)<br>*p* = 0.946; *q* = 0.985	0.0004 (-0.003, 0.003)<br>*p* = 0.805; *q* = 0.876	**Non-linear[1#]**<br>***p* < 0.001; *q* < 0.001**<br>**(Fig. S.7.1b)**	–	–
	Total road density (per 1000 m/km²; 500-m buffer)	-0.052 (-1.523, 1.420)<br>*p* = 0.945; *q* = 0.985	0.0002 (-0.003, 0.003)<br>*p* = 0.876; *q* = 0.876	-0.228 (-0.645, 0.189)<br>*p* = 0.285; q = 0.285	–	–
	Major road density (per 1000 m/km²; 500-m buffer)	-0.067 (-1.539, 1.405)<br>*p* = 0.929; *q* = 0.985	0.0003 (-0.003, 0.003)<br>*p* = 0.854; *q* = 0.876	**Non-linear[1]**<br>***p* = 0.044; *q* = 0.073**<br>**(Fig. S.7.3)**	–	–
	Distance to nearest busy road (per 100 m)	-0.043 (-1.514, 1.429)<br>*p* = 0.955; *q* = 0.985	0.0002 (-0.003, 0.003)<br>*p* = 0.872; *q* = 0.876	0.092 (-0.042, 0.225)<br>*p* = 0.180; *q* = 0.225	–	–
Diastolic BP (mmHg)	NO_2 (ppb)	-0.295 (-1.194, 0.605)<br>*p* = 0.521; *q* = 0.521	-0.001 (-0.003, 0.001)<br>*p* = 0.229; *q* = 0.283	-0.324 (-0.567, -0.082)<br>***p* = 0.009; *q* = 0.023**	–	–
	$PM_{2.5}$ (μg/m³)	-0.303 (-1.202, 0.595)<br>*p* = 0.508; *q* = 0.521	-0.001 (-0.003, 0.001)<br>*p* = 0.283; *q* = 0.283	0.411 (0.176, 0.646)<br>***p* < 0.001; *q* < 0.001**	–	–
	Total road density (per 1000 m/km²; 500-m buffer)	-0.318 (-1.218, 0.582)<br>*p* = 0.489; *q* = 0.521	-0.001 (-0.003, 0.001)<br>*p* = 0.227; *q* = 0.283	-0.083 (-0.333, 0.168)<br>*p* = 0.518; *q* = 0.648	–	–
	Major road density (per 1000 m/km²; 500-m buffer)	-0.324 (-1.224, 0.576)<br>*p* = 0.481; *q* = 0.521	-0.001 (-0.003, 0.001)<br>*p* = 0.233; *q* = 0.283	-0.122 (-0.415, 0.170)<br>*p* = 0.412; *q* = 0.648	–	–
	Distance to nearest busy road (per 100 m)	-0.320 (-1.220, 0.580)<br>*p* = 0.486; *q* = 0.521	-0.001 (-0.003, 0.001)<br>*p* = 0.241; *q* = 0.283	-0.002 (-0.082, 0.078)<br>*p* = 0.968; *q* = 0.968	–	–
HDL-C (mmol/L)	NO_2 (ppb)	0.015 (-0.018, 0.048)<br>*p* = 0.380; *q* = 0.392	0.0002 (0.0001, 0.0003)<br>***p* < 0.001; *q* < 0.001**	-0.002 (-0.011, 0.007)<br>*p* = 0.645; *q* = 0.903	–	–
	$PM_{2.5}$ (μg/m³)	0.014 (-0.019, 0.048)<br>*p* = 0.392; *q* = 0.392	0.0002 (0.0001, 0.0003)<br>***p* < 0.001; *q* < 0.001**	-0.006 (-0.015, 0.002)<br>*p* = 0.149; *q* = 0.373	–	–
	Total road density (per 1000 m/km²; 500-m buffer)	0.015 (-0.018, 0.048)<br>*p* = 0.376; *q* = 0.392	0.0002 (0.0001, 0.0003)<br>***p* < 0.001; *q* < 0.001**	-0.012 (-0.022, -0.003)<br>***p* = 0.009; *q* = 0.045**	–	Met
	Major road density (per 1000 m/km²; 500-m buffer)	0.015 (-0.018, 0.048)<br>*p* = 0.386; *q* = 0.392	0.0002 (0.0001, 0.0003)<br>***p* < 0.001; *q* < 0.001**	-0.001 (-0.012, 0.010)<br>*p* = 0.866; *q* = 0.903	–	–

CMB health outcome	Environmental indicator	Total walking participation & CMB adj. for environmental indicator	Total walking time & CMB adj. for environmental indicator	Environmental indicator & CMB adj. for both total walking variables	JS: walking participation	JS: walking time
	Distance to nearest busy road (per 100 m)	0.015 (-0.018, 0.048) *p* = 0.384; *q* = 0.392	0.0002 (0.0001,0.0003) ***p* < 0.001; *q* < 0.001**	0.0001 (-0.003, 0.003) *p* = 0.903; q = 0.903	—	Met
LDL-C (mmol/L)	NO_2 (ppb)	-0.044 (-0.118, 0.031) *p* = 0.253; *q* = 0.259	0.0002 (0.000, 0.0004) ***p* = 0.002; *q* = 0.003**	-0.007 (-0.026, 0.012) *p* = 0.463; *q* = 0.579	—	—
	$PM_{2.5}$ (μg/m³)	-0.043 (-0.117, 0.032) *p* = 0.259; *q* = 0.259	0.0002 (0.000, 0.0004) ***p* = 0.002; *q* = 0.003**	0.021 (0.003, 0.039) ***p* = 0.022;** *q* = 0.110	—	—
	Total road density (per 1000 m/km²; 500-m buffer)	-0.041 (-0.112, 0.031) *p* = 0.247; *q* = 0.259	0.0002 (0.000, 0.0004) ***p* = 0.003; *q* = 0.003**	-0.004 (-0.023, 0.016) *p* = 0.706; *q* = 0.706	—	Met
	Major road density (per 1000 m/km²; 500-m buffer)	-0.044 (-0.118, 0.031) *p* = 0.252; *q* = 0.259	0.0002 (0.000, 0.0004) ***p* = 0.002; *q* = 0.003**	0.014 (-0.009, 0.037) *p* = 0.223; *q* = 0.558	—	—
	Distance to nearest busy road (per 100 m)	-0.044 (-0.119, 0.030) *p* = 0.243; *q* = 0.259	0.0002 (0.0001, 0.0004) ***p* = 0.002; *q* = 0.003**	-0.003 (-0.009, 0.003) *p* = 0.359; *q* = 0.579	—	Met
Triglycerides (mmol/L)	NO_2 (ppb)	0.980 (0.930, 1.032) *p* = 0.435; *q* = 0.473	0.9999 (0.9998, 0.9999) ***p* = 0.009; *q* = 0.015**	0.999 (0.986, 1.011) *p* = 0.828; *q* = 0.828	—	—
	$PM_{2.5}$ (μg/m³)	0.980 (0.930, 1.032) *p* = 0.438; *q* = 0.473	0.9998 (0.9997, 0.9999) ***p* = 0.011; *q* = 0.015**	1.005 (0.993, 1.017) *p* = 0.428; *q* = 0.535	—	—
	Total road density (per 1000 m/km²; 500-m buffer)	0.980 (0.930, 1.032) *p* = 0.443; *q* = 0.473	0.9998 (0.9997, 0.9999) ***p* = 0.018; *q* = 0.018**	1.023 (1.010, 1.037) ***p* < 0.001; *q* < 0.001**	—	Met
	Major road density (per 1000 m/km²; 500-m buffer)	0.981 (0.931, 1.034) *p* = 0.473; *q* = 0.473	0.9998 (0.9997, 0.9999) ***p* = 0.011; *q* = 0.015**	1.021 (1.006, 1.037) ***p* = 0.007; *q* = 0.018**	—	—
	Distance to nearest busy road (per 100 m)	0.979 (0.930, 1.032) *p* = 0.431; *q* = 0.473	0.9998 (0.9997, 0.9999) ***p* = 0.012; *q* = 0.015**	0.998 (0.994, 1.002) *p* = 0.357; *q* = 0.535	—	Met
HbA1c (mmol/mol)	NO_2 (ppb)	0.986 (0.974, 0.998) ***p* = 0.023; *q* = 0.029**	0.9999 (0.9999, 1.000) *p* = 0.100; *q* = 0.101	1.003 (0.999, 1.007) *p* = 0.112; *q* = 0.280	—	—
	$PM_{2.5}$ (μg/m³)	0.986 (0.974, 0.998) ***p* = 0.025; *q* = 0.029**	0.9999 (0.9999, 1.000) *p* = 0.100; *q* = 0.101	1.001 (0.997, 1.005) *p* = 0.609; *q* = 0.748	—	—
	Total road density (per 1000 m/km²; 500-m buffer)	0.986 (0.974, 0.998) ***p* = 0.025; *q* = 0.029**	0.9999 (0.9999, 1.000) *p* = 0.101; *q* = 0.101	1.001 (0.997, 1.004) *p* = 0.748; *q* = 0.748	—	—

CMB health outcome	Environmental indicator	Total walking participation & CMB adj. for environmental indicator	Total walking time & CMB adj. for environmental indicator	Environmental indicator & CMB adj. for both total walking variables	JS: walking participation	JS: walking time
	Major road density (per 1000 m/km²; 500-m buffer)	0.986 (0.974, 0.998) **p = 0.026; q = 0.029**	0.9999 (0.9999, 1.000) p = 0.101; q = 0.101	1.002 (0.998, 1.006) p = 0.377; q = 0.628	—	—
	Distance to nearest busy road (per 100 m)	0.986 (0.974, 0.999) **p = 0.029; q = 0.029**	0.9999 (0.9999, 1.000) p = 0.077; q = 0.101	**Non-linear[1#] p < 0.001; q < 0.001 (Fig. S.7.1d)**	—	—

C. Environmental indicator–walking associations

Environmental indicator	Participation in total walking (yes/no)				Non-zero total walking time (min/week)			
	OR	95% CI	p	q	e^b	95% CI	p	q
NO_2 (ppb)	1.018	0.963, 1.076	0.535	0.790	0.988	0.967, 1.009	0.249	0.311
$PM_{2.5}$ (μg/m³)	0.962	0.911, 1.015	0.156	0.720	0.979	0.959, 0.999	**0.042**	0.105
Total road density (per 1000 m/km²; 200-m buffer)	1.023	0.981, 1.066	0.288	0.720	0.985	0.969, 1.001	0.064	0.107
Major road density (per 1000 m/km²; 200-m buffer)	0.988	0.939, 1.039	0.632	0.790	0.995	0.975, 1.015	0.622	0.622
Distance to nearest busy road (per 100 m)	1.000	0.981, 1.018	0.974	0.974	1.010	1.003, 1.017	**0.006**	**0.030**

D. CMB health models including total walking

CMB health outcome	Environmental indicator	Total walking participation & CMB adj. for environmental indicator	Total walking time & CMB adj. for environmental indicator	Environmental indicator & CMB adj. for both total walking variables	JS: walking participation	JS: walking time
Waist circumference (cm)	NO_2 (ppb)	-1.731 (-2.825, -0.637) **p = 0.002; q = 0.002**	-0.006 (-0.008, -0.004) **p < 0.001; q < 0.001**	0.046 (-0.241, 0.334) p = 0.752; q = 0.752	—	—
	$PM_{2.5}$ (μg/m³)	-1.724 (-2.817, -0.630) **p = 0.002; q = 0.002**	-0.006 (-0.008, -0.004) **p < 0.001; q < 0.001**	0.290 (0.002, 0.578) **p = 0.049;** q = 0.245	—	—
	Total road density (per 1000 m/km²; 200-m buffer)	-1.741 (-2.836, -0.647) **p = 0.002; q = 0.002**	-0.006 (-0.008, -0.004) **p < 0.001; q < 0.001**	0.085 (-0.127, 0.296) p = 0.433; q = 0.722	—	—

CMB health outcome	Environmental indicator	Total walking participation & CMB adj. for environmental indicator	Total walking time & CMB adj. for environmental indicator	Environmental indicator & CMB adj. for both total walking variables	JS: walking participation	JS: walking time
	Major road density (per 1000 m/km²; 200-m buffer)	-1.728 (-2.822, -0.634)<br>***p* = 0.002; *q* = 0.002**	-0.006 (-0.008, -0.004)<br>***p* < 0.001; *q* < 0.001**	-0.045 (-0.308, 0.218)<br>*p* = 0.736; *q* = 0.752	—	—
	Distance to nearest busy road (per 100 m)	-1.742 (-2.836, -0.648)<br>***p* = 0.002; *q* = 0.002**	-0.006 (-0.008, -0.004)<br>***p* < 0.001; *q* < 0.001**	-0.063 (-0.161, 0.035)<br>*p* = 0.210; *q* = 0.525	—	Met
Systolic BP (mmHg)	NO_2 (ppb)	0.090 (-1.383, 1.562)<br>*p* = 0.905; *q* = 0.955	0.0003 (-0.003, 0.003)<br>*p* = 0.845; *q* = 0.845	**Non-linear[1#]**<br>***p* < 0.001; *q* < 0.001**<br>**(Fig. S.7.1a)**	—	—
	$PM_{2.5}$ (μg/m³)	0.054 (-1.419, 1.526)<br>*p* = 0.943; *q* = 0.955	0.0004 (-0.003, 0.003)<br>*p* = 0.784; *q* = 0.845	**Non-linear[1#]**<br>***p* < 0.001; *q* < 0.001**<br>**(Fig. S.7.1b)**	—	—
	Total road density (per 1000 m/km²; 200-m buffer)	0.042 (-1.432, 1.516)<br>*p* = 0.955; *q* = 0.955	0.0004 (-0.003, 0.003)<br>*p* = 0.803; *q* = 0.845	0.054 (-0.232, 0.340)<br>*p* = 0.712; *q* = 0.712	—	—
	Major road density (per 1000 m/km²; 200-m buffer)	0.054 (-1.419, 1.527)<br>*p* = 0.943; *q* = 0.955	0.0004 (-0.003, 0.003)<br>*p* = 0.817; *q* = 0.845	-0.249 (-0.606, 0.107)<br>*p* = 0.170; *q* = 0.283	—	—
	Distance to nearest busy road (per 100 m)	0.064 (-1.409, 1.538)<br>*p* = 0.932; *q* = 0.955	0.0003 (-0.003, 0.003)<br>*p* = 0.835; *q* = 0.845	0.058 (-0.076, 0.193)<br>*p* = 0.395; *q* = 0.494	—	—
Diastolic BP (mmHg)	NO_2 (ppb)	-0.209 (-1.110, 0.691)<br>*p* = 0.649; *q* = 0.649	-0.001 (-0.003, 0.001)<br>*p* = 0.230; *q* = 0.292	-0.349 (-0.580, -0.118)<br>***p* = 0.003; *q* = 0.008**	—	—
	$PM_{2.5}$ (μg/m³)	-0.224 (-1.124, 0.677)<br>*p* = 0.626; *q* = 0.649	-0.001 (-0.003, 0.001)<br>*p* = 0.292; *q* = 0.292	0.422 (0.189, 0.655)<br>***p* < 0.001; *q* < 0.001**	—	—
	Total road density (per 1000 m/km²; 200-m buffer)	-0.231 (-1.133, 0.671)<br>*p* = 0.616; *q* = 0.649	-0.001 (-0.003, 0.001)<br>*p* = 0.248; *q* = 0.292	0.002 (-0.171, 0.174)<br>*p* = 0.984; *q* = 0.984	—	—
	Major road density (per 1000 m/km²; 200-m buffer)	-0.231 (-1.132, 0.671)<br>*p* = 0.616; *q* = 0.649	-0.001 (-0.003, 0.001)<br>*p* = 0.248; *q* = 0.292	-0.027 (-0.242, 0.188)<br>*p* = 0.805; *q* = 0.984	—	—
	Distance to nearest busy road (per 100 m)	-0.236 (-1.138, 0.666)<br>*p* = 0.608; *q* = 0.649	-0.001 (-0.003, 0.001)<br>*p* = 0.257; *q* = 0.292	-0.024 (-0.104, 0.056)<br>*p* = 0.559; *q* = 0.932	—	—
HDL-C (mmol/L)	NO_2 (ppb)	0.015 (-0.018, 0.049)<br>*p* = 0.363; *q* = 0.367	0.0002 (0.0001, 0.0003)<br>***p* < 0.001; *q* < 0.001**	-0.001 (-0.010, 0.007)<br>*p* = 0.801; *q* = 0.946	—	—

CMB health outcome	Environmental indicator	Total walking participation & CMB adj. for environmental indicator	Total walking time & CMB adj. for environmental indicator	Environmental indicator & CMB adj. for both total walking variables	JS: walking participation	JS: walking time
	$PM_{2.5}$ (μg/m³)	0.015 (-0.018, 0.048)<br>*p* = 0.367; *q* = 0.367	0.0002 (0.0001, 0.0003)<br>***p* < 0.001; *q* < 0.001**	-0.006 (-0.014, 0.003)<br>*p* = 0.184; *q* = 0.460	—	—
	Total road density (per 1000 m/km²; 200-m buffer)	0.016 (-0.017, 0.050)<br>*p* = 0.332; *q* = 0.367	0.0002 (0.0001, 0.0003)<br>***p* < 0.001; *q* < 0.001**	-0.007 (-0.013, -0.0004)<br>***p* = 0.036;** *q* = 0.180	—	—
	Major road density (per 1000 m/km²; 200-m buffer)	0.015 (-0.018, 0.048)<br>*p* = 0.365; *q* = 0.367	0.0002 (0.0001, 0.0003)<br>***p* < 0.001; *q* < 0.001**	-0.004 (-0.011, 0.004)<br>*p* = 0.380; *q* = 0.633	—	—
	Distance to nearest busy road (per 100 m)	0.015 (-0.018, 0.048)<br>*p* = 0.364; *q* = 0.367	0.0002 (0.0001,0.0003)<br>***p* < 0.001; *q* < 0.001**	0.0001 (-0.003, 0.003)<br>*p* = 0.946; *q* = 0.946	—	Met
LDL-C (mmol/L)	NO_2 (ppb)	-0.041 (-0.115, 0.034)<br>*p* = 0.282; *q* = 0.282	0.0002 (0.000, 0.0004)<br>***p* = 0.002; *q* = 0.002**	0.003 (-0.003, 0.008)<br>*p* = 0.278; *q* = 0.695	—	—
	$PM_{2.5}$ (μg/m³)	-0.041 (-0.112, 0.033)<br>*p* = 0.281; *q* = 0.282	0.0002 (0.000, 0.0004)<br>***p* = 0.002; *q* = 0.002**	0.019 (0.001, 0.036)<br>***p* = 0.036;** *q* = 0.180	—	—
	Total road density (per 1000 m/km²; 200-m buffer)	-0.041 (-0.115, 0.034)<br>*p* = 0.282; *q* = 0.282	0.0002 (0.000, 0.0004)<br>***p* = 0.002; *q* = 0.002**	-0.004 (-0.017, 0.010)<br>*p* = 0.584; *q* = 0.730	—	—
	Major road density (per 1000 m/km²; 200-m buffer)	-0.041 (-0.116, 0.033)<br>*p* = 0.275; *q* = 0.282	0.0002 (0.000, 0.0004)<br>***p* = 0.002; *q* = 0.002**	-0.002 (-0.019, 0.015)<br>*p* = 0.792; *q* = 0.792	—	—
	Distance to nearest busy road (per 100 m)	-0.042 (-0.116, 0.033)<br>*p* = 0.270; *q* = 0.282	0.0002 (0.0001, 0.0004)<br>***p* = 0.002; *q* = 0.002**	-0.002 (-0.008, 0.004)<br>*p* = 0.429; *q* = 0.715	—	Met
Triglycerides (mmol/L)	NO_2 (ppb)	0.985 (0.935, 1.037)<br>*p* = 0.561; *q* = 0.593	1.000 (1.000, 1.000)<br>***p* = 0.010; *q* = 0.013**	1.001 (0.989, 1.013)<br>*p* = 0.910; *q* = 0.910	—	—
	$PM_{2.5}$ (μg/m³)	0.985 (0.935, 1.038)<br>*p* = 0.567; *q* = 0.593	0.9998 (0.9997, 0.9999)<br>***p* = 0.013; *q* = 0.013**	1.008 (0.996, 1.020)<br>*p* = 0.197; *q* = 0.248	—	—
	Total road density (per 1000 m/km²; 200-m buffer)	0.983 (0.933, 1.036)<br>*p* = 0.531; *q* = 0.593	0.9998 (0.9997, 0.9999)<br>***p* = 0.012; *q* = 0.013**	1.010 (1.001, 1.019)<br>***p* = 0.038;** *q* = 0.120	—	—
	Major road density (per 1000 m/km²; 200-m buffer)	0.986 (0.936, 1.039)<br>*p* = 0.593; *q* = 0.593	0.9998 (0.9997, 0.9999)<br>***p* = 0.010; *q* = 0.013**	1.012 (1.000, 1.023)<br>***p* = 0.048;** *q* = 0.120	—	—
	Distance to nearest busy road (per 100 m)	0.984 (0.934, 1.037)<br>*p* = 0.555; *q* = 0.593	0.9998 (0.9997, 0.9999)<br>***p* = 0.012; *q* = 0.013**	0.997 (0.993, 1.001)<br>*p* = 0.198; *q* = 0.248	—	Met

CMB health outcome	Environmental indicator	Total walking participation & CMB adj. for environmental indicator	Total walking time & CMB adj. for environmental indicator	Environmental indicator & CMB adj. for both total walking variables	JS: walking participation	JS: walking time
HbA1c (mmol/mol)	NO_2 (ppb)	0.986 (0.974, 0.998) **p = 0.027; q = 0.035**	0.9999 (0.9999, 1.000) p = 0.091; q = 0.091	1.004 (1.001, 1.008) **p = 0.019; q = 0.048**	—	—
	$PM_{2.5}$ (μg/m^3)	0.986 (0.974, 0.999) **p = 0.030; q = 0.035**	0.9999 (0.9999, 1.000) p = 0.089; q = 0.091	1.002 (0.998, 1.005) p = 0.381; q = 0.635	—	—
	Total road density (per 1000 m/km^2; 200-m buffer)	0.986 (0.974, 0.999) **p = 0.030; q = 0.035**	0.9999 (0.9999, 1.000) p = 0.085; q = 0.091	1.000 (0.998, 1.003) p = 0.976; q = 0.976	—	—
	Major road density (per 1000 m/km^2; 200-m buffer)	0.986 (0.974, 0.999) **p = 0.030; q = 0.035**	0.9999 (0.9999, 1.000) p = 0.084; q = 0.091	1.000 (0.997, 1.003) p = 0.832; q = 0.976	—	—
	Distance to nearest busy road (per 100 m)	0.987 (0.975, 0.999) **p = 0.035; q = 0.035**	0.9999 (0.9999, 1.000) p = 0.069; q = 0.091	**Non-linear[1#] p < 0.001; q < 0.001 (Fig. S.7.1d)**	—	—

Notes: The joint-significance criterion was considered met when both the environmental indicator–walking association and the corresponding walking–CMB association adjusted for the environmental indicator had FDR-adjusted q-values < 0.05. A met JS criterion indicates a cross-sectional pattern of component associations consistent with a hypothesized behavioral pathway; it does not establish temporal ordering, an indirect effect or causal mediation. The environmental indicator–CMB association adjusted for walking is reported descriptively and should not be interpreted as a direct causal effect; it was not used to determine the JS criterion. Participation in total walking was modelled using a binomial GAMM with logit link; non-zero total walking time was modelled using a Gamma GAMM with logarithmic link. For waist circumference, systolic and diastolic BP, HDL-C and LDL-C, CMB-model estimates are regression coefficients (b). For triglycerides and HbA1c, estimates are exponentiated regression coefficients (e^b). $PM_{2.5}$, NO_2 and distance to the nearest busy road were not redefined by residential buffer; across sensitivity analyses, the buffer size refers to road-density measures and buffer-based neighborhood covariates. Road density was divided by 1000 and distance to the nearest busy road by 100, consistent with the source tables. CI, confidence interval; CMB, cardiometabolic; BP, blood pressure; HDL-C, high-density lipoprotein cholesterol; LDL-C, low-density lipoprotein cholesterol; HbA1c, glycated hemoglobin. adj., adjusted. p, p-value; q, Benjamini–Hochberg false discovery rate (FDR)-adjusted p-value. Bold p-values indicate p < 0.05; bold q-values indicate q < 0.05.[1] Pooled p-value for the smooth term obtained using a D1 multivariate Wald test based on Rubin's multiple-imputation combining rules.

[#]The shapes of the associations of NO_2, $PM_{2.5}$ with systolic BP, and of distance to nearest busy road with HbA1c were virtually identical across the three spatial scales of neighborhood-level covariates. Hence, here we present only those derived from the 1-km buffer models.

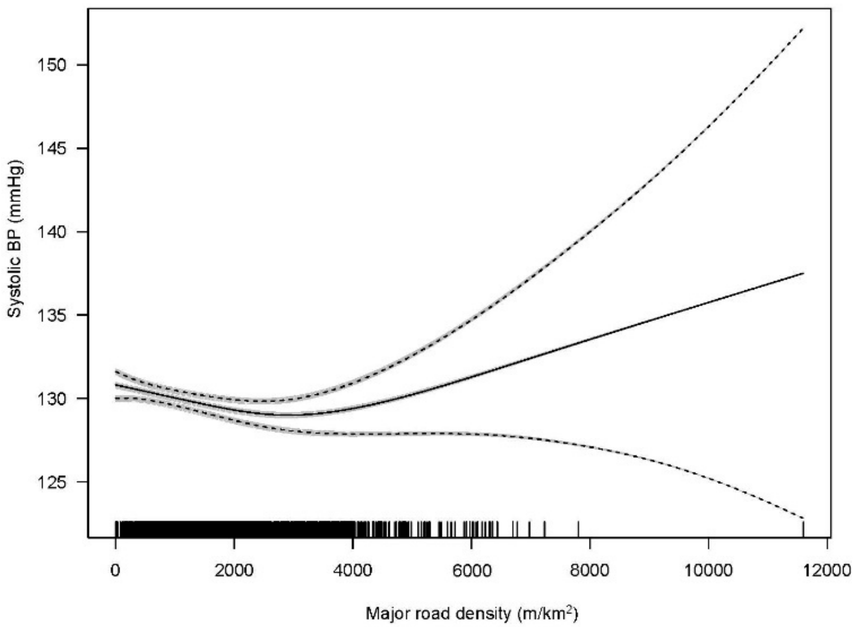

Figure S.7.3. Total walking-adjusted estimated non-linear associations of major road density (500-m buffer) with systolic blood pressure (did not meet the FDR-adjusted significance level, q < 0.05).

Table S.7.4. Spatial-scale sensitivity analyses of associations involving walking for transport used to evaluate hypothesized behavioral relationships between environmental indicators and cardiometabolic health.

A. Environmental indicator–walking associations

**Environmental indicator**	**Participation in walking for transport (yes/no)**				**Non-zero walking-for-transport frequency (times/week)**			
	**OR**	**95% CI**	***p***	***q***	**e^b**	**95% CI**	***p***	***q***
NO_2 (ppb)	1.090	1.035, 1.148	**0.001**	**0.003**	1.007	0.984, 1.029	0.568	0.568
$PM_{2.5}$ (μg/m³)	1.114	1.058, 1.174	**<0.001**	**<0.001**	1.018	0.993, 1.044	0.158	0.480
Total road density (per 1000 m/km²; 500-m buffer)	1.064	1.006, 1.125	**0.029**	**0.048**	1.007	0.983, 1.032	0.565	0.568
Major road density (per 1000 m/km²; 500-m buffer)	1.040	0.975, 1.109	0.233	0.233	0.981	0.952, 1.010	0.192	0.480
Distance to nearest busy road (per 100 m)	0.981	0.963, 0.999	**0.038**	**0.048**	1.004	0.995, 1.012	0.372	0.568

B. CMB health models including walking for transport

**CMB health outcome**	**Environmental indicator**	**Walking for transport participation & CMB adj. for environmental indicator**	**Walking-for-transport frequency & CMB adj. for environmental indicator**	**Environmental indicator & CMB adj. for both walking for transport variables**	**JS: walking participation**	**JS: walking frequency**
Waist circumference (cm)	NO_2 (ppb)	-2.097 (-3.606, -0.589)<br>***p* = 0.006; *q* = 0.006**	0.041 (-0.286, 0.367)<br>*p* = 0.807; *q* = 0.823	0.101 (-0.209, 0.411)<br>*p* = 0.522; *q* = 0.522	Met	—
	$PM_{2.5}$ (μg/m³)	-2.135 (-3.642, -0.627)<br>***p* = 0.006; *q* = 0.006**	0.037 (-0.289, 0.364)<br>*p* = 0.823; *q* = 0.823	0.383 (0.084, 0.682)<br>***p* = 0.012;** *q* = 0.060	Met	—
	Total road density (per 1000 m/km²; 500-m buffer)	-2.114 (-3.622, -0.606)<br>***p* = 0.006; *q* = 0.006**	0.043 (-0.283, 0.370)<br>*p* = 0.795; *q* = 0.823	0.287 (-0.028, 0.602)<br>*p* = 0.074; *q* = 0.180	Met	—
	Major road density (per 1000 m/km²; 500-m buffer)	-2.100 (-3.609, -0.592)<br>***p* = 0.006; *q* = 0.006**	0.044 (-0.282, 0.371)<br>*p* = 0.790; *q* = 0.823	0.146 (-0.223, 0.516)<br>*p* = 0.438; *q* = 0.522	—	—
	Distance to nearest busy road (per 100 m)	-2.121 (-3.629, -0.613)<br>***p* = 0.006; *q* = 0.006**	0.046 (-0.281, 0.372)<br>*p* = 0.783; *q* = 0.823	-0.083 (-0.185, 0.018)<br>*p* = 0.108; *q* = 0.180	Met	—
Systolic BP (mmHg)	NO_2 (ppb)	-0.386 (-2.431, 1.658)<br>*p* = 0.711; *q* = 0.711	0.001 (-0.438, 0.437)<br>*p* = 0.998; *q* = 0.998	**Non-linear[1#]**<br>***p* < 0.001; *q* < 0.001**<br>**(Fig. S.7.2a)**	—	—

CMB health outcome	Environmental indicator	Walking for transport participation & CMB adj. for environmental indicator	Walking-for-transport frequency & CMB adj. for environmental indicator	Environmental indicator & CMB adj. for both walking for transport variables	JS: walking participation	JS: walking frequency
	$PM_{2.5}$ (μg/m³)	-0.476 (-2.521, 1.569) *p* = 0.648; *q* = 0.711	-0.008 (-0.446, 0.429) *p* = 0.971; *q* = 0.998	**Non-linear[1#]** ***p* < 0.001; *q* < 0.001** **(Fig. S.7.2b)**	—	—
	Total road density (per 1000 m/km²; 500-m buffer)	-0.423 (-2.468, 1.623) *p* = 0.685; *q* = 0.711	-0.007 (-0.445, 0.430) *p* = 0.974; *q* = 0.998	-0.225 (-0.641, 0.191) *p* = 0.289; *q* = 0.289	—	—
	Major road density (per 1000 m/km²; 500-m buffer)	-0.415 (-2.461, 1.631) *p* = 0.691; *q* = 0.711	0.012 (-0.449, 0.426) *p* = 0.958; *q* = 0.998	**Non-linear[1]** ***p* = 0.045;** *q* = 0.075 **(Fig. S.7.4)**	—	—
	Distance to nearest busy road (per 100 m)	-0.407 (-2.452, 1.639) *p* = 0.697; *q* = 0.711	-0.010 (-0.448, 0.427) *p* = 0.963; *q* = 0.998	0.091 (-0.043, 0.224) *p* = 0.184; *q* = 0.230	—	—
Diastolic BP (mmHg)	NO_2 (ppb)	-0.345 (-1.585, 0.896) *p* = 0.586; *q* = 0.586	0.067 (-0.201, 0.335) *p* = 0.625; *q* = 0.669	-0.324 (-0.567, -0.082) ***p* = 0.009; *q* = 0.023**	—	—
	$PM_{2.5}$ (μg/m³)	-0.443 (-1.683, 0.796) *p* = 0.483; *q* = 0.586	0.058 (-0.210, 0.327) *p* = 0.669; *q* = 0.669	0.422 (0.187, 0.657) ***p* <0.001; *q* < 0.001**	—	—
	Total road density (per 1000 m/km²; 500-m buffer)	-0.374 (-1.616, 0.867) *p* = 0.554; *q* = 0.586	0.063 (-0.206, 0.331) *p* = 0.646; *q* = 0.669	-0.069 (-0.320, 0.181) *p* = 0.588; *q* = 0.735	—	—
	Major road density (per 1000 m/km²; 500-m buffer)	-0.371 (-1.613, 0.870) *p* = 0.558; *q* = 0.586	0.061 (-0.207, 0.330) *p* = 0.655; *q* = 0.669	-0.111 (-0.404, 0.181) *p* = 0.456; *q* = 0.735	—	—
	Distance to nearest busy road (per 100 m)	-0.384 (-1.626, 0.858) *p* = 0.545; *q* = 0.586	0.063 (-0.205, 0.332) *p* = 0.643; *q* = 0.669	-0.005 (-0.084, 0.075) *p* = 0.912; *q* = 0.912	—	—
HDL-C (mmol/L)	NO_2 (ppb)	0.060 (0.014, 0.106) ***p* = 0.010; *q* = 0.010**	-0.004 (-0.014, 0.006) *p* = 0.436; *q* = 0.443	-0.003 (-0.012, 0.006) *p* = 0.525; *q* = 0.644	Met	—
	$PM_{2.5}$ (μg/m³)	0.061 (0.015, 0.107) ***p* = 0.009; *q* = 0.010**	-0.004 (-0.014, 0.006) *p* = 0.443; *q* = 0.443	-0.009 (-0.017, 0.0003) *p* = 0.057; *q* = 0.143	Met	—
	Total road density (per 1000 m/km²; 500-m buffer)	0.062 (0.016, 0.108) ***p* = 0.008; *q* = 0.010**	-0.004 (-0.014, 0.006) *p* = 0.424; *q* = 0.443	-0.015 (-0.024, -0.006) ***p* = 0.002; *q* = 0.010**	Met	—
	Major road density (per 1000 m/km²; 500-m buffer)	0.060 (0.014, 0.106) ***p* = 0.010; *q* = 0.010**	-0.004 (-0.014, 0.006) *p* = 0.427; *q* = 0.443	-0.003 (-0.014, 0.008) *p* = 0.644; *q* = 0.644	—	—

CMB health outcome	Environmental indicator	Walking for transport participation & CMB adj. for environmental indicator	Walking-for-transport frequency & CMB adj. for environmental indicator	Environmental indicator & CMB adj. for both walking for transport variables	JS: walking participation	JS: walking frequency
	Distance to nearest busy road (per 100 m)	0.060 (0.014, 0.106)<br>***p* = 0.010; *q* = 0.010**	-0.004 (-0.014, 0.006)<br>*p* = 0.428; *q* = 0.443	0.001 (-0.002, 0.004)<br>*p* = 0.642; *q* = 0.644	Met	—
LDL-C (mmol/L)	NO_2 (ppb)	-0.065 (-0.167, 0.037)<br>*p* = 0.212; *q* = 0.212	0.025 (0.003, 0.047)<br>***p* = 0.025; *q* = 0.027**	-0.008 (-0.027, 0.011)<br>*p* = 0.402; *q* = 0.539	—	—
	$PM_{2.5}$ (μg/m³)	-0.069 (-0.171, 0.033)<br>*p* = 0.183; *q* = 0.212	0.025 (0.003, 0.047)<br>***p* = 0.027; *q* = 0.027**	0.019 (0.001,0.037)<br>***p* = 0.034; *q* = 0.170**	—	—
	Total road density (per 1000 m/km²; 500-m buffer)	-0.065 (-0.167, 0.037)<br>*p* = 0.212; *q* = 0.212	0.025 (0.003, 0.047)<br>***p* = 0.026; *q* = 0.027**	-0.006 (-0.025, 0.014)<br>*p* = 0.562; *q* = 0.562	—	—
	Major road density (per 1000 m/km²; 500-m buffer)	-0.067 (-0.169, 0.035)<br>*p* = 0.196; *q* = 0.212	0.025 (0.003, 0.048)<br>***p* = 0.025; *q* = 0.027**	0.014 (-0.009, 0.037)<br>*p* = 0.246; *q* = 0.539	—	—
	Distance to nearest busy road (per 100 m)	-0.067 (-0.169, 0.035)<br>*p* = 0.199; *q* = 0.212	0.025 (0.003, 0.047)<br>***p* = 0.025; *q* = 0.027**	-0.002 (-0.009, 0.004)<br>*p* = 0.431; *q* = 0.539	—	—
Triglycerides (mmol/L)	NO_2 (ppb)	0.944 (0.879, 1.013)<br>*p* = 0.107; *q* = 0.107	1.000 (0.985, 1.015)<br>*p* = 0.970; *q* = 0.993	1.000 (0.987, 1.013)<br>*p* = 0.994; *q* = 0.994	—	—
	$PM_{2.5}$ (μg/m³)	0.942 (0.878, 1.010)<br>*p* = 0.095; *q* = 0.107	1.000 (0.985, 1.015)<br>*p* = 0.973; *q* = 0.993	1.008 (0.996, 1.020)<br>*p* = 0.205; *q* = 0.265	—	—
	Total road density (per 1000 m/km²; 500-m buffer)	0.939 (0.875, 1.008)<br>*p* = 0.080; *q* = 0.107	1.000 (0.985, 1.016)<br>*p* = 0.977; *q* = 0.993	1.026 (1.012, 1.039)<br>***p* < 0.001; *q* < 0.001**	—	—
	Major road density (per 1000 m/km²; 500-m buffer)	0.940 (0.876, 1.009)<br>*p* = 0.086; *q* = 0.107	1.000 (0.985, 1.016)<br>*p* = 0.977; *q* = 0.993	1.023 (1.007, 1.038)<br>***p* = 0.004; *q* = 0.010**	—	—
	Distance to nearest busy road (per 100 m)	0.942 (0.878, 1.010)<br>*p* = 0.095; *q* = 0.107	1.000 (0.985, 1.015)<br>*p* = 0.993; *q* = 0.993	0.997 (0.993, 1.001)<br>*p* = 0.212; *q* = 0.265	—	—
HbA1c (mmol/mol)	NO_2 (ppb)	1.002 (0.985, 1.019)<br>*p* = 0.833; *q* = 0.833	0.998 (0.995, 1.002)<br>*p* = 0.424; *q* = 0.448	1.003 (0.999, 1.007)<br>*p* = 0.113; *q* = 0.283	—	—
	$PM_{2.5}$ (μg/m³)	1.002 (0.985, 1.019)<br>*p* = 0.820; *q* = 0.833	0.999 (0.995, 1.002)<br>*p* = 0.435; *q* = 0.448	1.001 (0.998, 1.005)<br>*p* = 0.512; *q* = 0.640	—	—
	Total road density (per 1000 m/km²; 500-m buffer)	1.002 (0.985, 1.019)<br>*p* = 0.817; *q* = 0.833	0.999 (0.995, 1.002)<br>*p* = 0.440; *q* = 0.448	1.001 (0.997, 1.005)<br>*p* = 0.640; *q* = 0.640	—	—

CMB health outcome	Environmental indicator	Walking for transport participation & CMB adj. for environmental indicator	Walking-for-transport frequency & CMB adj. for environmental indicator	Environmental indicator & CMB adj. for both walking for transport variables	JS: walking participation	JS: walking frequency
	Major road density (per 1000 m/km²; 500-m buffer)	1.002 (0.985, 1.019) *p* = 0.826; *q* = 0.833	0.999 (0.995, 1.002) *p* = 0.448; *q* = 0.448	1.002 (0.998, 1.007) *p* = 0.330; *q* = 0.550	—	—
	Distance to nearest busy road (per 100 m)	1.003 (0.986, 1.020) *p* = 0.758; *q* = 0.833	0.998 (0.995, 1.002) *p* = 0.417; *q* = 0.448	**Non-linear[1#]** ***p* < 0.001; *q* < 0.001** **(Fig. S.7.2d)**	—	—

C. Environmental indicator–walking associations

Environmental indicator	Participation in walking for transport (yes/no)				Non-zero walking-for-transport frequency (times/week)			
	OR	95% CI	*p*	*q*	e^b	95% CI	*p*	*q*
NO_2 (ppb)	1.100	1.047, 1.155	**<0.001**	**<0.001**	1.005	0.984, 1.026	0.641	0.801
$PM_{2.5}$ (μg/m³)	1.145	1.089, 1.204	**<0.001**	**<0.001**	1.019	0.994, 1.044	0.134	0.335
Total road density (per 1000 m/km²; 200-m buffer)	1.051	1.011, 1.092	**0.012**	**0.015**	1.000	0.983, 1.017	0.972	0.972
Major road density (per 1000 m/km²; 200-m buffer)	1.042	0.995, 1.091	0.079	0.079	0.984	0.964, 1.004	0.118	0.335
Distance to nearest busy road (per 100 m)	0.974	0.957, 0.992	**0.004**	**0.007**	1.002	0.994, 1.010	0.621	0.801

D. CMB health models including walking for transport

CMB health outcome	Environmental indicator	Walking for transport participation & CMB adj. for environmental indicator	Walking-for-transport frequency & CMB adj. for environmental indicator	Environmental indicator & CMB adj. for both walking for transport variables	JS: walking participation	JS: walking frequency
Waist circumference (cm)	NO_2 (ppb)	-2.081 (-3.586, -0.577) ***p* = 0.007; *q* = 0.007**	0.046 (-0.283, 0.374) *p* = 0.785; *q* = 0.810	0.086 (-0.204, 0.377) *p* = 0.560; q = 0.700	Met	—
	$PM_{2.5}$ (μg/m³)	-2.131 (-3.634, -0.628) ***p* = 0.005; *q* = 0.007**	0.040 (-0.288, 0.368) *p* = 0.810; *q* = 0.810	0.377 (0.085, 0.669) ***p* = 0.011;** *q* = 0.055	Met	—
	Total road density (per 1000 m/km²; 200-m buffer)	-2.088 (-3.592, -0.584) ***p* = 0.007; *q* = 0.007**	0.048 (-0.281, 0.376) *p* = 0.776; *q* = 0.810	0.102 (-0.111, 0.315) *p* = 0.347; *q* = 0.578	Met	—

CMB health outcome	Environmental indicator	Walking for transport participation & CMB adj. for environmental indicator	Walking-for-transport frequency & CMB adj. for environmental indicator	Environmental indicator & CMB adj. for both walking for transport variables	JS: walking participation	JS: walking frequency
	Major road density (per 1000 m/km²; 200-m buffer)	-2.064 (-3.568, -0.559)<br>***p* = 0.007; *q* = 0.007**	0.046 (-0.283, 0.374)<br>*p* = 0.785; *q* = 0.810	-0.025 (-0.290, 0.241)<br>*p* = 0.856; *q* = 0.856	—	—
	Distance to nearest busy road (per 100 m)	-2.105 (-3.609, -0.601)<br>***p* = 0.006; *q* = 0.007**	0.049 (-0.279, 0.377)<br>*p* = 0.771; *q* = 0.810	-0.080 (-0.179, 0.019)<br>*p* = 0.114; *q* = 0.285	Met	—
Systolic BP (mmHg)	NO_2 (ppb)	-0.386 (-2.419, 1.648)<br>*p* = 0.710; *q* = 0.710	0.028 (-0.409, 0.465)<br>*p* = 0.909; *q* = 0.940	**Non-linear[1#]**<br>***p* < 0.001; *q* < 0.001**<br>**(Fig. S.7.2a)**	—	—
	$PM_{2.5}$ (μg/m³)	-0.516 (-2.551, 1.518)<br>*p* = 0.619; *q* = 0.710	0.020 (-0.418, 0.457)<br>*p* = 0.929; *q* = 0.940	**Non-linear[1#]**<br>***p* < 0.001; *q* < 0.001**<br>**(Fig. S.7.2b)**	—	—
	Total road density (per 1000 m/km²; 200-m buffer)	-0.486 (-2.520, 1.549)<br>*p* = 0.640; *q* = 0.710	0.025 (-0.413, 0.462)<br>*p* = 0.912; *q* = 0.940	0.057 (-0.229, 0.343)<br>*p* = 0.696; *q* = 0.696	—	—
	Major road density (per 1000 m/km²; 200-m buffer)	-0.430 (-2.465, 1.605)<br>*p* = 0.679; *q* = 0.710	0.017 (-0.421, 0.454)<br>*p* = 0.940; *q* = 0.940	-0.247 (-0.603, 0.109)<br>*p* = 0.175; *q* = 0.292	—	—
	Distance to nearest busy road (per 100 m)	-0.449 (-2.483, 1.586)<br>*p* = 0.666; *q* = 0.710	0.022 (-0.415, 0.460)<br>*p* = 0.921; *q* = 0.940	0.057 (-0.077, 0.191)<br>*p* = 0.405; *q* = 0.506	—	—
Diastolic BP (mmHg)	NO_2 (ppb)	-0.288 (-1.525, 0.948)<br>*p* = 0.648; *q* = 0.648	0.078 (-0.189, 0.345)<br>*p* = 0.568; *q* = 0.617	-0.348 (-0.580, -0.117)<br>***p* = 0.003; *q* = 0.008**	—	—
	$PM_{2.5}$ (μg/m³)	-0.426 (-1.662, 0.811)<br>*p* = 0.500; *q* = 0.648	0.068 (-0.199, 0.335)<br>*p* = 0.617; *q* = 0.617	0.431 (0.197, 0.665)<br>***p* < 0.001; *q* < 0.001**	—	—
	Total road density (per 1000 m/km²; 200-m buffer)	-0.348 (-1.586, 0.889)<br>*p* = 0.581; *q* = 0.648	0.075 (-0.192, 0.342)<br>*p* = 0.581; *q* = 0.617	0.003 (-0.169, 0.176)<br>*p* = 0.969; *q* = 0.969	—	—
	Major road density (per 1000 m/km²; 200-m buffer)	-0.343 (-1.581, 0.895)<br>*p* = 0.587; *q* = 0.648	0.074 (-0.193, 0.342)<br>*p* = 0.585; *q* = 0.617	-0.024 (-0.239, 0.191)<br>*p* = 0.826; *q* = 0.969	—	—
	Distance to nearest busy road (per 100 m)	-0.360 (-1.598, 0.877)<br>*p* = 0.569; *q* = 0.648	0.076 (-0.191, 0.343)<br>*p* = 0.578; *q* = 0.617	-0.026 (-0.106, 0.054)<br>*p* = 0.525; *q* = 0.875	—	—
HDL-C (mmol/L)	NO_2 (ppb)	0.060 (0.014, 0.106)<br>***p* = 0.011; *q* = 0.011**	-0.004 (-0.014, 0.006)<br>*p* = 0.450; *q* = 0.463	-0.002 (-0.011, 0.006)<br>*p* = 0.595; *q* = 0.671	Met	—

CMB health outcome	Environmental indicator	Walking for transport participation & CMB adj. for environmental indicator	Walking-for-transport frequency & CMB adj. for environmental indicator	Environmental indicator & CMB adj. for both walking for transport variables	JS: walking participation	JS: walking frequency
	$PM_{2.5}$ (μg/m³)	0.061 (0.015, 0.107)<br>***p* = 0.009; *q* = 0.011**	-0.004 (-0.014, 0.006)<br>*p* = 0.463; *q* = 0.463	-0.008 (-0.017, 0.001)<br>*p* = 0.068; *q* = 0.170	Met	—
	Total road density (per 1000 m/km²; 200-m buffer)	0.061 (0.015, 0.107)<br>***p* = 0.009; *q* = 0.011**	-0.004 (-0.014, 0.006)<br>*p* = 0.436; *q* = 0.463	-0.007 (-0.014, -0.001)<br>***p* = 0.021;** *q* = 0.105	Met	—
	Major road density (per 1000 m/km²; 200-m buffer)	0.060 (0.014, 0.106)<br>***p* = 0.010; *q* = 0.011**	-0.004 (-0.014, 0.006)<br>*p* = 0.434; *q* = 0.463	-0.004 (-0.012, 0.004)<br>*p* = 0.300; *q* = 0.500	—	—
	Distance to nearest busy road (per 100 m)	0.060 (0.014, 0.106)<br>***p* = 0.011; *q* = 0.011**	-0.004 (-0.014, 0.006)<br>*p* = 0.445; *q* = 0.463	0.001 (-0.002, 0.004)<br>*p* = 0.671; *q* = 0.671	Met	—
LDL-C (mmol/L)	NO_2 (ppb)	-0.060 (-0.162, 0.042)<br>*p* = 0.251; *q* = 0.251	0.024 (0.002, 0.046)<br>***p* = 0.030; *q* = 0.033**	-0.011 (-0.029, 0.007)<br>*p* = 0.230; *q* = 0.575	—	—
	$PM_{2.5}$ (μg/m³)	-0.065 (-0.167, 0.037)<br>*p* = 0.209; *q* = 0.251	0.024 (0.002, 0.046)<br>***p* = 0.033; *q* = 0.033**	0.017 (-0.001, 0.035)<br>*p* = 0.059; *q* = 0.295	—	—
	Total road density (per 1000 m/km²; 200-m buffer)	-0.060 (-0.162, 0.042)<br>*p* = 0.246; *q* = 0.251	0.024 (0.002, 0.046)<br>***p* = 0.032; *q* = 0.033**	-0.005 (-0.018, 0.009)<br>*p* = 0.515; *q* = 0.651	—	—
	Major road density (per 1000 m/km²; 200-m buffer)	-0.061 (-0.163, 0.041)<br>*p* = 0.243; *q* = 0.251	0.024 (0.002, 0.046)<br>***p* = 0.032; *q* = 0.033**	-0.002 (-0.019, 0.015)<br>*p* = 0.784; *q* = 0.784	—	—
	Distance to nearest busy road (per 100 m)	-0.062 (-0.164, 0.040)<br>*p* = 0.231; *q* = 0.251	0.024 (0.002, 0.046)<br>***p* = 0.031; *q* = 0.033**	-0.002 (-0.008, 0.004)<br>*p* = 0.521; *q* = 0.651	—	—
Triglycerides (mmol/L)	NO_2 (ppb)	0.945 (0.880, 1.014)<br>*p* = 0.113; *q* = 0.113	1.000 (0.985, 1.016)<br>*p* = 0.995; *q* = 0.996	1.002 (0.990, 1.014)<br>*p* = 0.707; *q* = 0.707	—	—
	$PM_{2.5}$ (μg/m³)	0.942 (0.878, 1.011)<br>*p* = 0.096; *q* = 0.113	1.000 (0.985, 1.016)<br>*p* = 0.996; *q* = 0.996	1.011 (0.999, 1.023)<br>*p* = 0.073; *q* = 0.122	—	—
	Total road density (per 1000 m/km²; 200-m buffer)	0.942 (0.878, 1.011)<br>*p* = 0.100; *q* = 0.113	1.000 (0.985, 1.016)<br>*p* = 0.965; *q* = 0.996	1.011 (1.001, 1.020)<br>***p* = 0.025;** *q* = 0.083	—	—
	Major road density (per 1000 m/km²; 200-m buffer)	0.942 (0.878, 1.011)<br>*p* = 0.096; *q* = 0.113	1.001 (0.985, 1.016)<br>*p* = 0.948; *q* = 0.996	1.013 (1.001, 1.024)<br>***p* = 0.033;** *q* = 0.083	—	—
	Distance to nearest busy road (per 100 m)	0.942 (0.878, 1.011)<br>*p* = 0.099; *q* = 0.113	1.000 (0.985, 1.016)<br>*p* = 0.969; *q* = 0.996	0.997 (0.993, 1.001)<br>*p* = 0.103; *q* = 0.129	—	—

CMB health outcome	Environmental indicator	Walking for transport participation & CMB adj. for environmental indicator	Walking-for-transport frequency & CMB adj. for environmental indicator	Environmental indicator & CMB adj. for both walking for transport variables	JS: walking participation	JS: walking frequency
HbA1c (mmol/mol)	NO_2 (ppb)	1.002 (0.985, 1.019) $p = 0.850$; $q = 0.850$	0.999 (0.995, 1.002) $p = 0.494$; $q = 0.512$	1.004 (1.001, 1.008) **$p = 0.018$; $q = 0.045$**	—	—
	$PM_{2.5}$ ($\mu g/m^3$)	1.002 (0.985, 1.019) $p = 0.820$; $q = 0.850$	0.999 (0.995, 1.002) $p = 0.503$; $q = 0.512$	1.002 (0.998, 1.005) $p = 0.316$; $q = 0.527$	—	—
	Total road density (per 1000 m/km^2; 200-m buffer)	1.002 (0.985, 1.019) $p = 0.799$; $q = 0.850$	0.999 (0.995, 1.002) $p = 0.512$; $q = 0.512$	1.000 (0.998, 1.002) $p = 0.994$; $q = 0.994$	—	—
	Major road density (per 1000 m/km^2; 200-m buffer)	1.002 (0.985, 1.020) $p = 0.794$; $q = 0.850$	0.999 (0.995, 1.002) $p = 0.509$; $q = 0.512$	1.000 (0.997, 1.003) $p = 0.827$; $q = 0.994$	—	—
	Distance to nearest busy road (per 100 m)	1.003 (0.986, 1.020) $p = 0.749$; $q = 0.850$	0.999 (0.995, 1.002) $p = 0.498$; $q = 0.512$	**Non-linear[1#] $p < 0.001$; $q < 0.001$ (Fig. S.7.2d)**	—	—

Notes: The joint-significance criterion was considered met when both the environmental indicator–walking association and the corresponding walking–CMB association adjusted for the environmental indicator had FDR-adjusted *q*-values < 0.05. A met JS criterion indicates a cross-sectional pattern of component associations consistent with a hypothesized behavioral pathway; it does not establish temporal ordering, an indirect effect or causal mediation. The environmental indicator–CMB association adjusted for walking is reported descriptively and should not be interpreted as a direct causal effect; it was not used to determine the JS criterion. Participation in walking for transport was modelled using a binomial GAMM with logit link; non-zero walking-for-transport frequency was modelled using a negative-binomial GAMM with logarithmic link. For waist circumference, systolic and diastolic BP, HDL-C and LDL-C, CMB-model estimates are regression coefficients (*b*). For triglycerides and HbA1c, estimates are exponentiated regression coefficients (e^b). $PM_{2.5}$, NO_2 and distance to the nearest busy road were not redefined by residential buffer; across sensitivity analyses, the buffer size refers to road-density measures and buffer-based neighborhood covariates. Road density was divided by 1000 and distance to the nearest busy road by 100, consistent with the source tables. CI, confidence interval; CMB, cardiometabolic; BP, blood pressure; HDL-C, high-density lipoprotein cholesterol; LDL-C, low-density lipoprotein cholesterol; HbA1c, glycated hemoglobin. adj., adjusted. *p*, *p*-value; *q*, Benjamini–Hochberg false discovery rate (FDR)-adjusted *p*-value. Bold *p*-values indicate $p < 0.05$; bold *q*-values indicate $q < 0.05$. [1] Pooled *p*-value for the smooth term obtained using a D1 multivariate Wald test based on Rubin's multiple-imputation combining rules.

[#]The shapes of the associations of NO_2, $PM_{2.5}$ with systolic BP, and of distance to nearest busy road with HbA1c were virtually identical across the three spatial scales of neighborhood-level covariates. Hence, here we present only those derived from the 1-km buffer models.

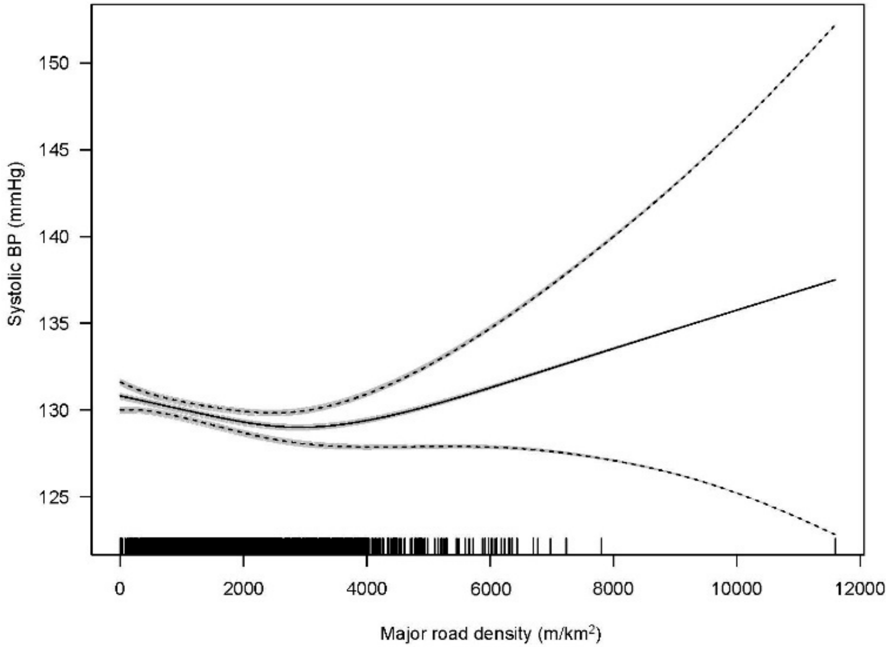

Figure S.7.4. Walking for transport-adjusted estimated non-linear associations of major road density (500-m buffer) with systolic blood pressure (did not meet the FDR-adjusted significance level, q < 0.05).
